# Synthesis and *In Vitro* Evaluation of Anticancer Activity of Fluorophenyl Derivatives of 1,3,4-Thiadiazole Against Estrogen-Dependent Breast Cancer

**DOI:** 10.3390/molecules30244744

**Published:** 2025-12-12

**Authors:** Sara Janowska, Anna Makuch-Kocka, Rafał Kurczab, Oleg M. Demchuk, Monika Wujec

**Affiliations:** 1 Department of Experimental and Clinical Pharmacology, Medical University of Lublin, 8b Jaczewskiego Street, 20-090 Lublin, Poland; 2 Department of Pharmacology, Medical University of Lublin, Radziwiłłowska 11 Street, 20-080 Lublin, Poland; anna.makuch-kocka@umlub.edu.pl; 3 Department of Medicinal Chemistry, Maj Institute of Pharmacology, Polish Academy of Sciences, 12 Smetna Street, 31-343 Kraków, Poland; kurczab@if-pan.krakow.pl; 4 Faculty of Medicine, The John Paul II Catholic University of Lublin, 1J Konstantynów Street, 20-708 Lublin, Poland; oleh.demchuk@kul.pl; 5 Department of Organic Chemistry, Medical University of Lublin, 4A Chodźki Street, 20-093 Lublin, Poland

**Keywords:** 1,3,4-Thiadiazole derivatives, aromatase inhibitors, estrogen-dependent breast cancer, cytotoxicity

## Abstract

Breast cancer remains the most frequently diagnosed malignant tumor among women worldwide, and the limited selectivity as well as the emerging resistance to currently used therapies highlight the need to search for new therapeutic compounds. Aromatase, a key enzyme in the estrogen biosynthesis pathway, represents a recognized molecular target in the treatment of hormone-dependent cancers. In this study, six new 1,3,4-thiadiazole derivatives containing two halogen-substituted aromatic rings were designed and synthesized as potential nonsteroidal aromatase inhibitors. The cytotoxic activity of the obtained compounds was evaluated against two breast cancer cell lines: MCF-7 (estrogen-dependent) and MDA-MB-231 (estrogen-independent). All tested compounds exhibited concentration-dependent cytotoxic activity against MCF-7 cells, with the strongest effects observed for compounds **A2**, **A3**, **B1**, and **B3** (IC_50_ ≈ 52–55 µM). In contrast, none of the tested compounds showed significant activity against MDA-MB-231 cells (IC_50_ > 100 µM), suggesting their selectivity toward estrogen-dependent cancer cells. Compound **B3**, identified as the most promising, was further subjected to in silico analyses. Molecular docking and molecular dynamics simulations revealed that **B3** occupies a binding site similar to that of the co-crystallized native inhibitor and forms interactions characteristic of strong aromatase inhibitors. The obtained results confirm a mechanism of action related to aromatase inhibition and indicate that fluorophenyl-substituted 1,3,4-thiadiazole derivatives represent a promising scaffold for the design of new, selective, and less toxic aromatase inhibitors.

## 1. Introduction

Breast cancer is currently the most frequently diagnosed malignant cancer in women worldwide. According to the World Health Organization, there were 2.3 million new cases of female breast cancer and 670,000 related deaths worldwide in 2022 [1]. Most currently used pharmacological treatments for this disease are associated with serious side effects, and their efficacy remains insufficient. These difficulties stem from the low selectivity of known anticancer drugs and the rapid rate at which cancer cells develop drug resistance.

One promising direction in the search for new drugs effective in treating cancer, particularly estrogen-dependent breast cancer, is aromatase inhibitors. In mammals, including humans, this enzyme is responsible for catalyzing the conversion of androgens into estrogens [2]. Consequently, aromatase becomes a key enzyme in the estrogen biosynthesis pathway, which plays a significant role in the development of hormone-dependent breast cancers. The link between aromatase and breast cancer arises from the fact that increased estrogen concentrations stimulate the growth of these cancers. Simultaneously, it has been observed that breast cancer cells exhibit increased expression of the aromatase enzyme, leading them to synthesize higher concentrations of estrogens than normal cells. For this reason, aromatase represents an interesting molecular target for new forms of pharmacotherapy, and aromatase inhibitors have become an important group of molecules with anticancer potential.

In 2009, a research team led by Ghosh determined the three-dimensional crystal structure of aromatase [3]. This significant discovery allowed for a better understanding of how the enzyme binds to its native ligand. This work revealed that the active site of aromatase contains non-polar, hydrophobic side chains that enable fitting the steroid backbone of the substrates. After elucidating the structure of the enzyme’s active site, it became possible to design drug molecules that—despite lacking a steroid structure—can bind to and block this enzyme.

From the perspective of organic synthesis and targeted pharmacotherapy design, a particularly interesting group is nonsteroidal aromatase inhibitors, also known as type II inhibitors [4]. This category includes drugs that have been or are used in clinical practice, such as fadrozole, vorozole, rogletimide, letrozole, and anastrozole. Studies indicate that molecules in this group most often bind non-covalently to the heme residue in the aromatase enzyme. Their attachment to the enzyme’s active center blocks the ability of native ligands, such as androgens, to bind. Inhibition of aromatase by nonsteroidal inhibitors is reversible [5].

Based on a literature review, it can be observed that nonsteroidal aromatase inhibitors almost always have two key chemical elements in their structure [5]. One is an azole moiety containing a built-in nitrogen atom that interacts with the iron atom in the heme of aromatase. Another important structural element is an extensive aryl part, which makes the nonsteroidal inhibitor molecule resemble the steroid ring characteristic of the natural substrate of the reaction catalyzed by the enzyme.

**Scheme 1:**
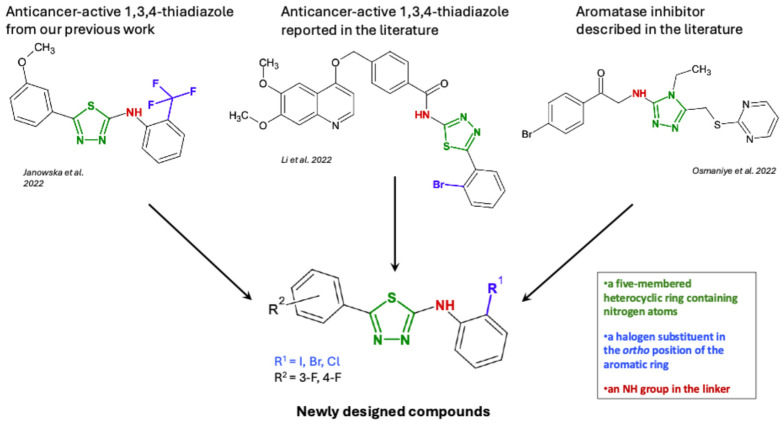
Rational design of the new molecule based on structural motifs reported in previous studies and active reference compounds [9,12,13].

Based on this data, we designed and synthesized new small-molecule compounds with anticancer potential. 1,3,4-Thiadiazole derivatives were chosen as the subject of research due to numerous reports indicating the anticancer activity of this group of heterocyclic compounds [6,7,8,9,10,11,12]. The mentioned heterocyclic structure contains two nitrogen atoms, which potentially enable interactions with the heme in the active side of aromatase. The synthesized derivatives differed in the structure of the substituents at the C2 and C5 positions, where phenyl groups with halogen atoms at various positions on the ring were introduced, which, according to the literature review, should favorably affect the affinity of these compounds for aromatase [5,13]. We presented the process of designing new molecules based on our earlier research and literature data in Scheme 1.

To evaluate the effect of the studied compounds on cell viability, experiments were performed using two human breast cancer cell lines: MCF-7 (estrogen-dependent) and MDA-MB-231 (triple-negative), as well as a normal human mammary fibroblast (HMF) line. The mechanism of action we propose for these compounds involves aromatase inhibition, which we decided to verify by performing molecular modeling studies of this enzyme.

## 2. Results and Discussion

### 2.1. Chemistry

**Scheme 2:**
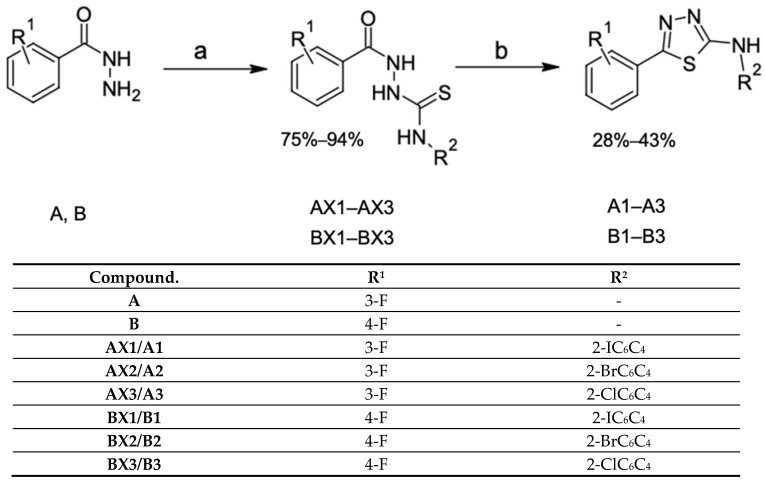
Synthesis of thiosemicarbazide and 1,3,4-thiadiazole derivatives. Reagents and conditions: (**a**) aryl isothiocyanates, 96% EtOH, reflux; (**b**) H_2_SO_4_ (conc), rt.

The title compounds **A1–A3** and **B1–B3** were obtained through the two-step synthesis presented in Scheme 2.

The reaction of 3-fluorobenzhydrazides and 4-fluorobenzhydrazides with aryl isothiocyanates (2-chlorophenyl, 2-bromophenyl, and 2-iodophenyl) afforded the corresponding 1,4-disubstituted thiosemicarbazides **AX1–AX3** and **BX1–BX3**. These compounds were subsequently treated with concentrated sulfuric acid at room temperature, which led to the formation of 1,3,4-thiadiazole derivatives.

The reaction yields ranged from 75 to 94% for thiosemicarbazides and 28–43% for 1,3,4-thiadiazoles **A1–A3** and **B1–B3**. The low yield in the synthesis of 1,3,4-thiadiazole derivatives via cyclization is related to the dehydration of the molecule under concentrated 98% sulfuric acid conditions. Under these conditions, partial degradation of the substrate occurs. Nevertheless, this method remains the most efficient for obtaining 1,3,4-thiadiazoles. In total, 12 compounds were obtained (6 thiosemicarbazides and 6 thiadiazoles), of which 11 had not been previously reported in the scientific literature.

The structures of all new compounds were confirmed by IR, ^1^H NMR, and ^13^C NMR spectroscopy.

- In the IR spectra of compounds **A1–A3** and **B1–B3**, absorption bands in the range of 697–762 cm^−1^ were observed, characteristic of the S-C bond present in the heterocyclic 1,3,4-thiadiazole system.
- The ^1^H NMR spectra showed characteristic chemical shifts for protons N1, N2, and N4, confirming the presence of the thiosemicarbazide scaffold. These protons appeared as three singlet signals in the range of 9.65–10.71 ppm.
- The ^1^H NMR spectra also confirmed the formation of thiadiazole derivatives. Protons N1, N2, and N4, characteristic of thiosemicarbazides, were absent in the spectra of 1,3,4-thiadiazoles. Instead, a signal corresponding to the N–H proton of the amino group was observed in the range of 9.80–10.10 ppm.
- The ^13^C NMR spectra showed signals consistent with the proposed structures of the compounds.
- In the ^13^C NMR spectra of compounds **A1**–**A3** and **B1**–**B3**, doublets with coupling constants characteristic of fluorine atoms bonded to carbon were observed. Doublets with coupling constants of 244.0–248.3 Hz were recorded, corresponding to carbons directly attached to fluorine. Additionally, doublets with coupling constants in the range of 21.0–23.8 Hz were observed, typical for carbons located one bond away from the fluorine atom. Doublets with coupling constants of 8.3–8.8 Hz were also detected, corresponding to carbons in the meta position relative to the fluorine substituent, as well as doublets with coupling constants of 2.9–3.1 Hz, characteristic of carbons situated in the para position with respect to the fluorine atom.
- The complete ^1^H NMR and ^13^C NMR spectra are provided in the Supplementary Materials.

The compound with CAS number 894227-09-9 (**BX3**) has been previously reported in the literature. However, its detailed physicochemical properties are not available, prompting us to provide a full characterization herein.

### 2.2. Evaluation of the Effects of Tested Compounds on Cell Viability—MTT Assay

To investigate how the tested compounds affect cell viability, experiments were performed using two human breast cancer cell lines—MDA-MB-231 and MCF-7 and human fibroblasts—HMF (normal cells). The MDA-MB-231 cell line represents a highly invasive, triple-negative breast cancer (TNBC) model, lacking estrogen receptor (ER), progesterone receptor (PR), and HER2 expression. In contrast, MCF-7 cells are estrogen receptor-positive (ER+), less invasive, and hormone-responsive, making them a well-established model for luminal-type breast cancer and hormone therapy research [14]. Anastrozole, an aromatase inhibitor, served as a reference drug. The MTT assay applied in this study relies on mitochondrial dehydrogenase enzymes to reduce a water-soluble orange tetrazolium salt (3-(4,5-dimethylthiazol-2-yl)-2,5-diphenyltetrazolium bromide, or MTT) into a dark blue, insoluble formazan compound. The amount of this formazan product formed is directly related to the mitochondrial activity of viable, metabolically active cells in the sample. The efficacy of the compounds was assessed based on IC_50_ values and analysis of the concentration-dependence of cell survival.

**Figure 1:**
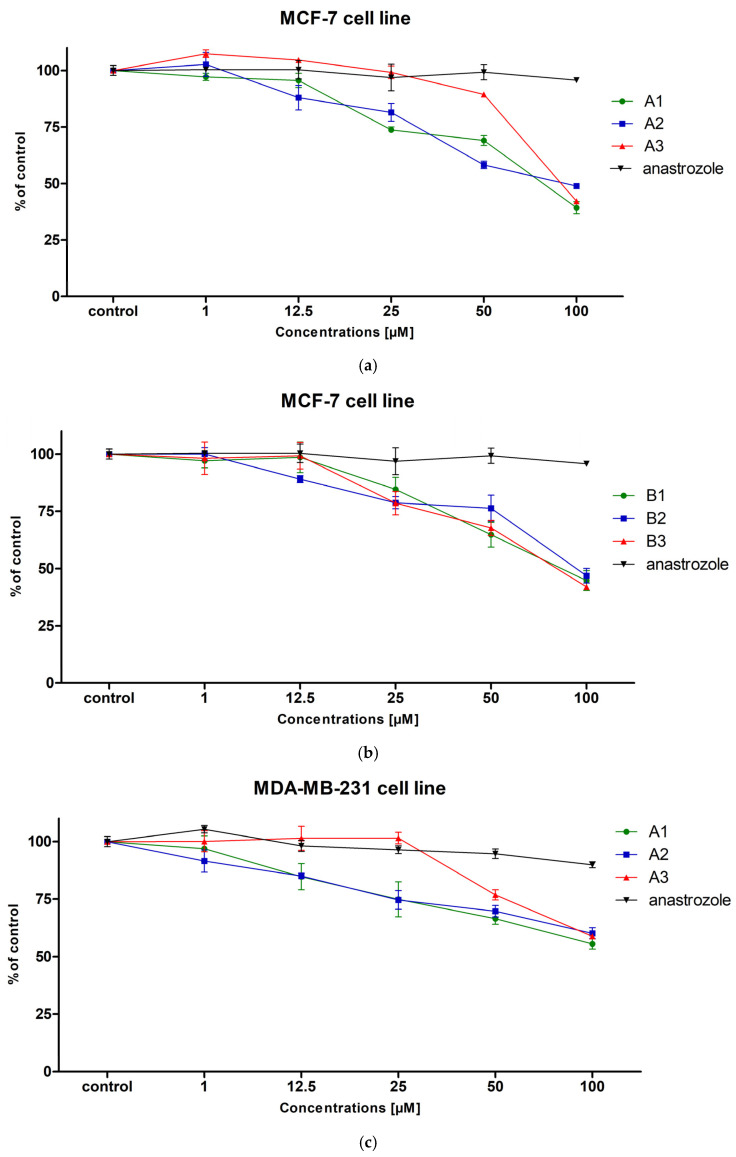

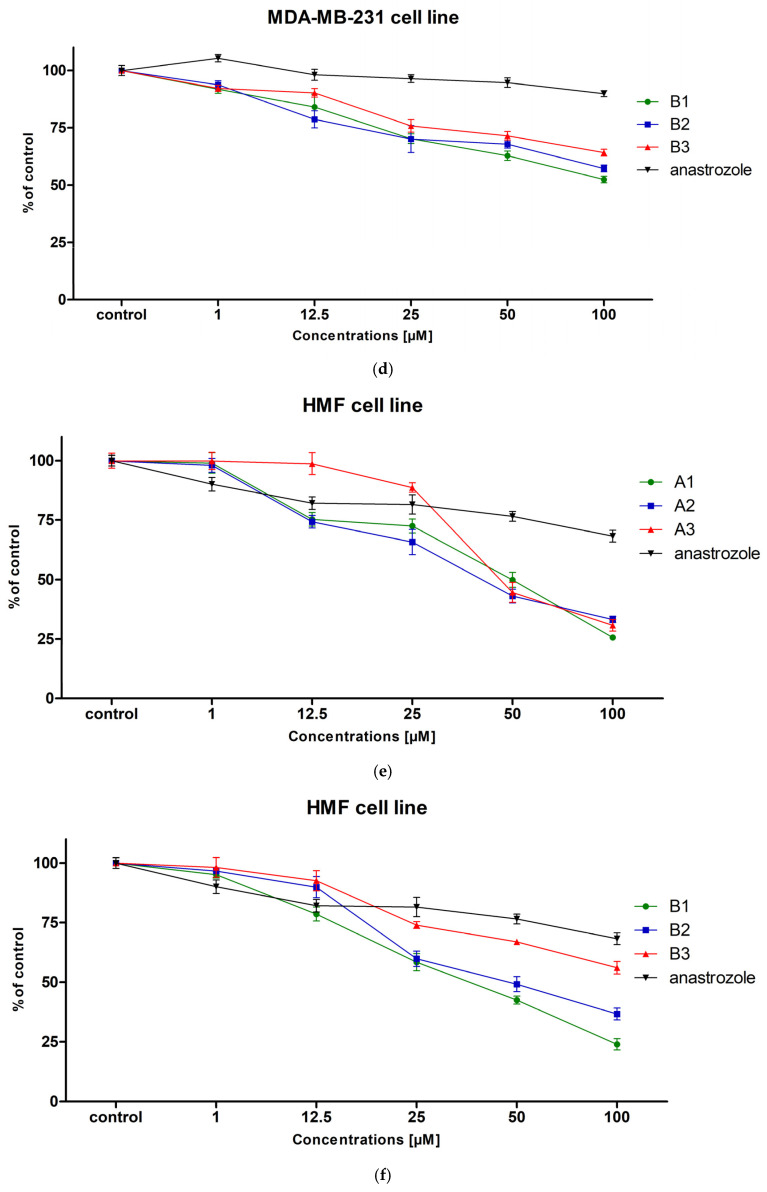
The viability of MCF-7 (**a**,**b**), MDA-MB-231 (**c**,**d**) and HMF (**e**,**f**) cells exposed to different concentrations of the tested compounds (**A1**–**A3**, **B1**–**B3**) for 24 h. Mean values ± SD from two independent experiments performed in quadruplicate are presented.

In the case of MCF-7 cells, all tested compounds showed cytotoxic activity in a concentration-dependent manner (Figure 1a,b). The strongest antitumor activity among all tested compounds was observed for compound **A2** (IC_50_ = 52.35 µM), as well as for compounds **A3** (IC_50_ = 54.81 µM), **B1** (IC_50_ = 53.9 µM) and **B3** (IC_50_ = 54.1 µM), which indicates their comparable efficacy against this breast cancer cell line. Compound **A1** showed slightly weaker activity (IC_50_ = 65.95 µM), while compound **B2** was characterized by the lowest activity in this group (IC_50_ = 82.48 µM). All compounds tested showed lower IC_50_ values (were more toxic) than anastrozole (IC_50_ > 100 µM).

In the case of the triple-negative breast cancer cell line MDA-MB-231, none of the tested compounds, similarly to anastrozole, achieved an IC_50_ value below 100 µM, indicating their low cytotoxic activity against this more aggressive and resistant cell line (Figure 1c,d). This may suggest that the tested compounds are not effective against cells with low expression of hormone receptors and HER2, which in turn may indicate a mechanism of action dependent on signaling pathways absent in this cell line. The selective activity of the tested compounds against the MCF-7 line may indicate a potential mechanism of action dependent on the presence of hormone receptors. However, further research is needed to confirm this mechanism.

To assess the safety of potential anticancer drug candidates, we analyzed their cytotoxic activity against the normal HMF cell line. The results showed that the compounds differed in their toxicity towards fibroblasts (Figure 1e,f). The highest IC_50_ value (over 100 µM) was observed for compound **B3**, indicating its low toxicity towards fibroblasts and a potentially favorable safety profile. Compounds **B1**, **B2**, and **A1** showed moderate cytotoxicity (IC_50_ = 45.58 µM, 41.8 µM, and 51.01 µM, respectively), whereas compounds **A2** and **A3** were significantly more toxic (IC_50_ = 36.46 µM and 36.9 µM), which may suggest their limited selectivity towards cancer cells. Based on these results, compound **B3** appears particularly promising in terms of safety, demonstrating minimal toxicity to normal cells while maintaining activity against MCF-7 cancer cells. These data indicate the need for further evaluation of the selectivity of the tested compounds, particularly in the context of their potential therapeutic applications.

From the perspective of the relationship between chemical structure and bioavailability/activity, the compounds in the studied series were characterized by very similar parameters. All molecules featured a heterocyclic ring containing nitrogen atoms in their structure, to which two aromatic systems were attached, each containing a halogen substituent. According to a previous literature review, molecules containing such structural elements can be potential aromatase inhibitors. This assumption was confirmed by the results of biological tests, where the cytotoxic potential of the investigated compounds was significantly higher against the estrogen-dependent MCF-7 cancer cell line than against the estrogen-independent MDA-MB-231 line.

The cytotoxic activity of all compounds in the series against cancer cells was very similar, which likely results from their comparable bioavailability parameters, related to factors such as lipophilicity and molecular size. At the same time, significant differences in cytotoxicity towards normal cell lines were observed within the series. Compounds **A1**–**A3**, possessing a fluorine atom in the meta position on the aromatic ring at position 5 relative to the aromatic system, exhibited higher toxicity towards fibroblasts. All these substances were characterized by lower IC_50_ values for normal cells (36.46–51.01 µM) than for MCF-7 cancer cells (52.35–65.95 µM). This relationship indicates that they are not good candidates for anticancer drugs. The IC50 values for all compounds are presented in Table 1.

In contrast, compounds **B1**–**B3**, possessing a fluorine atom in the para position, were characterized by higher IC_50_ values for fibroblasts, ranging from 41.8 µM to >100 µM. Among them, only compound **B3** showed lower cytotoxicity towards normal cells (IC_50_ > 100 µM) than towards estrogen-dependent cancer cells (IC_50_ = 54.1 µM). This least toxic molecule, being a potential drug candidate, differs from the others by the presence of a chlorine atom in the ortho position on the phenyl ring at position 2 relative to the 1,3,4-thiadiazole system. Compounds with an iodine (**B1**) or bromine (**B2**) atom in this position exhibited higher toxicity towards normal cell lines than towards cancer cell lines.

In summary, the analysis indicates that among the investigated compounds, the chlorinated derivative (**B3**) exhibits the most favorable safety and biological activity profile, making it the most promising candidate for further research.

In our previous study, we demonstrated the promising activity of 1,3,4-thiadiazole derivatives against the estrogen-dependent breast cancer cell line MCF-7. The most active compound, 2-(2-trifluoromethylphenylamino)-5-(3-methoxyphenyl)-1,3,4-thiadiazole, exhibited an IC_50_ value of 49.6 µM [9]. In the present series, the best-performing compound showed an IC_50_ value of 54.1 µM, which is comparable to the most favorable activities observed previously.

Referring to other studies on anticancer compounds acting as aromatase inhibitors, it can be noted that small heterocyclic systems containing nitrogen atoms, such as triazole and imidazole derivatives, display high potential [5]. In our research, we investigate compounds that additionally contain a sulfur atom within the heterocyclic ring, alongside nitrogen atoms. A structural element that favors activity is the connection of the heterocyclic system with a substituted phenyl ring. Substitution in the para position appears particularly advantageous, which is partially confirmed by our findings—the best-performing compound contains a fluorine atom in this position of the phenyl ring [5].

Substituents that clearly enhance the ability of the molecule to inhibit aromatase are halogen atoms. For example, Osmaniye et al. described new pyrimidinotriazole derivatives, among which the compound bearing a bromine atom in the para position of the phenyl ring exhibited the most promising activity against the MCF-7 cell line, with an IC_50_ value of 1.573 μM [13]. Similarly, Çetiner et al. synthesized a series of imidazole derivatives, among which the compound with a para-substituted phenyl ring demonstrated activity toward the MCF-7 cell line at the level of IC_50_ = 7.16 µM [15].

Another structural element favoring activity is the connection of the heterocyclic system with the phenyl ring via a linker that allows rotation. Particularly beneficial appears to be the presence of an NH group within such a linker, as it positively influences the molecule’s ability to inhibit aromatase. This element is present in all the compounds presented in the current series, as well as in all active molecules described in the articles cited in our discussion [9,13,15].

### 2.3. Molecular Docking Study

**Figure 2:**
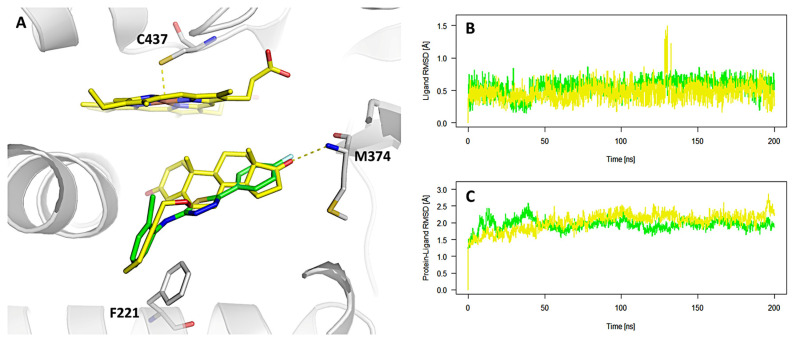
Binding mode of compound **B3** (green) in the active site of the aromatase enzyme. For comparison, the binding position and orientation of the co-crystallized inhibitor (yellow) are also shown (**A**). Panels (**B**,**C**) present the RMSD trajectories calculated from 200 ns long MD simulations for **B3** and the native inhibitor (**B**), as well as for their respective enzyme–ligand complexes (**C**).

Among all compounds synthesized in this study, compound **B3** was identified as the most promising candidate for further development. Therefore, **B3** was selected for detailed molecular modeling to elucidate its interaction mechanism with the aromatase enzyme. Molecular docking analysis of compound **B3** within the active site of aromatase revealed three potential binding modes (Figure S37A). To assess their relative stability, 200 ns molecular dynamics (MD) simulations were performed for each docking pose. The average RMSD values calculated for the ligand heavy atoms were **0.56 ± 0.12 Å, 0.97 ± 0.47 Å**, and **1.24 ± 0.27 Å** for the first, second, and third binding modes, respectively. These results clearly indicate that the first binding mode is the most stable conformation throughout the 200 ns simulation (as illustrated in Figure 1, compared with the native inhibitor). In this complex, compound **B3** occupies nearly the same binding site as the native co-crystallized inhibitor. The complex is stabilized primarily by π–π stacking interactions between the 2-Cl–Ar fragment of **B3** and Phe221, as well as by a polar dipole–dipole interaction between the C–F group of the 4F–Ar fragment and Met374. These interactions are consistent with those typically observed for potent aromatase inhibitors, suggesting that **B3** effectively mimics key molecular features required for enzyme binding and inhibition. Additionally, the results of the 200 ns MD simulations indicate that the stability of the B3–enzyme complex is comparable to that of the native inhibitor (**0.47 ± 0.14 Å**), which is clearly reflected in the RMSD profiles presented in Figure 2B,C.

## 3. Materials and Methods

### 3.1. Chemistry

All the substances were purchased from Sigma-Aldrich (Munich, Germany) and were used without further purification. The ^1^H and ^13^C NMR spectra were recorded on the BrukerAvance 600 (Bruker BioSpin GmbH, Rheinstetten, Germany). IR spectra were recorded by Nicolet 6700 spectrometer (Thermo Scientific, Philadelphia, PA, USA). The HPLC and MS analyses were performed on a Shimadzu LC-MS Q-Tof 9030 ESI (Kyoto, Japan) instrument equipped with a column Dr. Maisch ReproSil-Pur Basic-C18 3 µm 100 mm (Dr. Maisch HPLC GmbH, Ammerbuch-Entingen, Germany), which was eluted at 40 °C by MeOH/H_2_O = 70/30 with a flow rate of 0.3 mL/min. The melting points were determined on the Stuart SMP50 melting point apparatus (Cole Parmer Ltd., Stone, UK) and are uncorrected. The purity of the compounds and the progress of the reaction were monitored by TLC aluminum sheet 60 F254 plates (Merck Co., Kenilworth, NJ, USA). We used the solvent system CHCl_3_/EtOH (10:1, *v*/*v*).

#### 3.1.1. Synthesis of Thiosemicarbazide Derivatives **AX1**–**AX3** and **BX1**–**BX3**

In a round-bottom flask, 5 mL of anhydrous ethanol was placed, and 0.001 mol of 3-fluorobenzhydrazide (compounds **AX1**–**AX3**) or 4-fluorobenzhydrazide (compounds **BX1**–**BX3**) was dissolved in it. The mixture was heated under reflux until a clear solution was obtained. Next, an equimolar amount of the appropriate aryl isothiocyanate* was added to the boiling mixture. The reaction mixture was then maintained under reflux for 1 h for **BX2**, **AX1**, and **AX2**; for 3 h for **BX1** and **BX3**; and for 15 h for **AX3**. Afterward, it was cooled to room temperature and left until the product had fully precipitated. The resulting solid was filtered off under reduced pressure. The product was then purified by washing with hot demineralized water, followed by washing with diethyl ether. The filtered solid was dried under vacuum.

* **AX1** and **BX1**—2-iodophenyl isothiocyanate; **AX2** and **BX2**—2-bromophenyl isothiocyanate; **AX3** and **BX3**—2-chlorophenyl isothiocyanate.

**1-(3-Fluorobenzoyl)-4-(2-iodophenyl)thiosemicarbazide (AX1)**

Yield 75%, m.p. 156–158 °C. Spectral data were as follows: ^1^H NMR (DMSO-d_6_) δ 7.00–7.11 (m, 1H, ArH), 7.31–7.47 (m, 3H, ArH), 7.53–7.60 (m, 1H, ArH), 7.82 (d, *J* = 7.9 Hz, 2H, ArH), 7.88 (d, *J* = 7.9 Hz, 1H, ArH), 9.69 (s, 1H, NH), 9.84 (s, 1H, NH), 10.71 (s, 1H, NH). ^13^C NMR (DMSO-d_6_) δ 94.88, 114.88 (d, J = 23.3 Hz), 118.72, 120.42 (d, *J =* 21.5 Hz), 123.09 (d, *J* = 30.1 Hz), 124.28, 126.10 (d, *J =* 28.9 Hz), 126.83, 129.68 (d, *J =* 37.4 Hz), 131.56 (d, *J =* 8.2 Hz), 133.72 (d, *J =* 7.2 Hz), 161.52, 163.46, 166.78.

**4-(2-Bromophenyl)-1-(3-fluorobenzoyl)thiosemicarbazide (AX2)**

Yield 84%, m.p. 148–149 °C. Spectral data were as follows: ^1^H NMR (600 MHz, DMSO-d_6_) δ 7.16–7.27 (m, 1H, ArH), 7.35–7.50 (m, 3H, ArH), 7.53–7.63 (m, 1H, ArH), 7.61–7.72 (m, 1H, ArH), 7.74–7.88 (m, 2H, ArH), 9.70 (s, 1H, NH), 9.88 (s, 1H, NH), 10.72 (s, 1H, NH). ^13^C NMR (DMSO-d_6_) δ 115.33 (d, *J =* 22.4 Hz), 119.21 (d, *J =* 21.1 Hz), 128.18, 128.79, 129.28, 130.92, 131.57, 132.89, 135.31, 138.74, 162.24 (d, *J =* 243.8 Hz), 165.47, 182.31.

**4-(2-Chlorophenyl)-1-(3-fluorobenzoyl)thiosemicarbazide (AX3)**

CAS 894227-03-3

Yield 86%, m.p. 149–150 °C. Spectral data were as follows: ^1^H NMR (MHz, DMSO-d_6_) δ 7.28–7.31 (m, 1H, ArH), 7.34–7.39 (m, 2H, ArH), 7.46 (td, *J =* 8.7, 8.2, 2.5 Hz, 1H, ArH), 7.50 (d, *J* = 8.0 Hz 1H, ArH), 7.56–7.59 (m, 1H, ArH), 7.81 (d, *J =* 7.4 Hz, 2H, ArH), 9.72 (s, 1H, NH), 9.91 (s, 1H, NH), 10.73 (s, 1H, NH). ^13^C NMR (DMSO-d_6_) δ 115.32 (d, *J =* 22.8 Hz), 119.23 (d, *J =* 20.8 Hz), 124.66, 127.56, 128.56, 129.78, 130.92, 131.55, 135.29, 137.30, 161.44, 163.05, 165.42, 182.43.

**1-(4-Fluorobenzoyl)-4-(2-iodophenyl)thiosemicarbazide (BX1)**

Yield 82%, m.p. 189–191 °C. Spectral data were as follows: ^1^H NMR (DMSO-d_6_) δ 7.03 (t, *J =* 8.4 Hz, 1H, ArH), 7.35 (t, *J =* 8.9 Hz, 3H, ArH), 7.39–7.42 (m, 1H, ArH), 7.87 (d, *J =* 7.9 Hz, 1H, ArH), 8.04–8.06, 9.66 (s, 1H, NH), 9.81 (s, 1H, NH), 10.64 (s, 1H, NH). ^13^C NMR (DMSO-d_6_) δ 100.46, 115.56, 115.71, 128.78, 129.55, 130.61, 131.27, 139.07, 142.00, 164.57, 165.53, 165.78, 182.13.

**4-(2-Bromophenyl)-1-(4-fluorobenzoyl)thiosemicarbazide (BX2)**

Yield 94%, m.p. 180–181 °C. Spectral data were as follows: ^1^H NMR (DMSO-d_6_) δ 7.18–7.25 (m, 1H, ArH), 7.34–7.38 (m, 2H, ArH), 7.38–7.49 (m, 2H, ArH), 7.62–7.72 (m, 1H, ArH), 8.04 (s, 2H, ArH), 9.68 (s, 1H, NH), 9.85 (s, 1H, NH), 10.65 (s, 1H, NH). ^13^C NMR (DMSO-d_6_) δ 115.66 (d, *J =* 21.9 Hz), 122.67, 128.15, 128.72, 129.49, 131.23, 131.41, 132.87, 138.74, 165.54, 165.62 (d, *J =* 24.5 Hz), 182.30.

**4-(2-Chlorophenyl)-1-(4-fluorobenzoyl)thiosemicarbazide (BX3)** [16]

CAS 894227-09-9.

Yield 87%, m.p. 180–182 °C. Spectral data were as follows: ^1^H NMR (DMSO-d_6_) δ 7.29 (t, *J* = 8.1 Hz, 1H, ArH), 7.34–7.37 (m, 3H, ArH), 7.41 (d, *J* = 8.0 Hz, 1H, ArH), 7.50 (d, J = 8.0 Hz, 1H, ArH), 8.03–8.05 (m, 2H, ArH), 9.70 (s, 1H, NH), 9.88 (s, 1H, NH), 10.66 (s, 1H, NH). ^13^C NMR (DMSO-d_6_) 115.59, 115.74, 127.53, 128.48, 129.48, 129.75, 131.21, 131.44, 131.89, 137.31, 164.79 (d, *J =* 249.7 Hz), 182.42

#### 3.1.2. Synthesis of 1,3,4-Thiadiazole Derivatives **A1**–**A3** and **B1**–**B3**

An amount of 0.2 g of the thiosemicarbazide derivatives obtained in the first stage was weighed and placed into conical flasks. To the powdered compound, 0.5 mL of concentrated sulfuric acid (H_2_SO_4_) was added dropwise with vigorous stirring. The reaction mixture was stirred with a metal spatula until the precipitate was completely dissolved, and then it was left to stand for 1 h at room temperature. Next, finely crushed ice was added to the flasks containing the reaction mixtures and stirred intensively. A solid precipitated from the solution, which—after the ice had completely melted—was filtered off under reduced pressure. The solid on the filter was washed with a small amount of demineralized water. The product was thoroughly dried using filter paper and then purified by recrystallization from isopropanol. In this way, the pure compounds **A1**–**A3** and **B1**–**B3** were obtained. The filtered solid was dried under vacuum.

**5-(3-Fluorophenyl)-2-(2-iodophenylamino)-1,3,4-thiadiazole (A1)**

Yield 38%, m.p. 156–158 °C. Spectral data were as follows: IR (cm^−1^) KBr: 2995 (NH), 2912 (CH aroma.), 1668 (C=N), 1534 (CH arom.), 764 (C-S). ^1^H NMR (DMSO-d_6_) δ 7.01 (t, *J =* 7.6 Hz, 1H, ArH), 7.28–7.39 (m, 1H, ArH), 7.46 (td, *J =* 7.6, 1.5 Hz, 1H, ArH), 7.49–7.58 (m, 1H, ArH), 7.63–7.68 (m, 2H, ArH), 7.77 (s, 1H, ArH), 7.95 (dd, *J =* 7.9, 1.4 Hz, 1H, ArH), 9.90 (s, 1H, NH). ^13^C NMR (DMSO-d_6_) δ 95.32, 113.39 (d, *J =* 23.8 Hz), 117.47 (d, *J =* 21.3 Hz), 123.46, 127.90, 129.99, 132.01 (d, *J =* 8.3 Hz), 132.99, 140.15, 162.87 (d, *J =* 244.7 Hz), 167.13. HRMS (ESI): *m*/*z* = calcd. 396.95459 (M^+^H^+^, C_14_ H_9_ N_3_ F S I), found 397.96134, diff. −1.332 ppm.

**2-(2-Bromophenylamino)-5-(3-fluorophenyl)-1,3,4-thiadiazole (A2)**

Yield 43%, m.p. 168–169 °C. Spectral data were as follows: IR (cm^−1^) KBr: 2994 (NH), 2912 (CH aroma.), 1594 (C=N), 1542 (CH arom.), 697 (C-S). ^1^H NMR (DMSO-d_6_) δ 7.10–7.11 (m, 1H, ArH), 7.32–7.38 (m, 1H, ArH), 7.42–7.47 (m, 1H, ArH), 7.54–7.58 (m, 1H, ArH), 7.66–7.72 (m, 3H, ArH), 8.09 (s, 1H, ArH), 9.98 (s, 1H, NH). ^13^C NMR (DMSO-d_6_) δ 113.48 (d, *J =* 23.6 Hz), 115.69, 117.48 (d, *J =* 21.1 Hz), 123.52, 124.18, 126.28, 129.06, 131.93 (d, *J =* 8.4 Hz), 132.93, 133.62, 162.82 (d, *J =* 244.7 Hz), 166.41. HRMS (ESI): *m*/*z* = calcd. 348.96846 (M^+^H^+^, C_14_ H_9_ N_3_ F S Br), found 349.97532, diff. −1.188 ppm.

**2-(2-Chlorophenylamino)-5-(3-fluorophenyl)-1,3,4-thiadiazole (A3)**

Yield 28%, m.p. 152–154 °C. Spectral data were as follows: IR (cm^−1^) KBr: 3431 (NH), 2994 (CH aroma.), 1593 (C=N), 1542 (CH arom.), 697 (C-S). ^1^H NMR (600 MHz, DMSO-d_6_) δ 7.12–7.18 (m, 1H, ArH), 7.32–7.43 (m, 2H, ArH), 7.50–7.60 (m, 2H, ArH), 7.66–7.74 (m, 2H, ArH), 8.29 (s, 1H, ArH), 10.10 (s, 1H, NH). ^13^C NMR (126 MHz, DMSO-d_6_) δ 113.57 (d, *J =* 23.4 Hz), 117.63 (d, *J =* 21.0 Hz), 122.84, 123.66 (d, *J =* 2.9 Hz), 124.03, 125.27, 128.52, 130.33, 132.04 (d, *J =* 8.4 Hz), 132.92 (d, *J =* 8.4 Hz), 162.88 (d, *J =* 244.7 Hz), 165.90. HRMS (ESI): *m*/*z* = calcd. 305.01897 (M^+^H^+^, C_14_ H_9_ N_3_ F S Cl), found 306.02629, diff. 0.128 ppm.

**5-(4-Fluorophenyl)-2-(2-iodophenylamino)-1,3,4-thiadiazole (B1)**

Yield 36%, m.p. 177–179 °C. Spectral data were as follows: IR (cm^−1^) KBr: 3192 (NH), 3015 (CH aroma.), 1602 (C=N), 1574 (CH arom.), 762 (C-S). ^1^H NMR (DMSO-d_6_) δ 6.99 (td, *J =* 7.6, 1.6 Hz, 1H, ArH), 7.31–7.38 (m, 2H, ArH), 7.43–7.48 (m, 1H, ArH), 7.76 (s, 1H, ArH), 7.84–7.90 (m, 2H, ArH), 7.94 (dd, *J =* 7.9, 1.5 Hz, 1H, ArH), 9.80 (s, 1H, NH). ^13^C NMR (DMSO-d_6_) δ 95.06, 116.84 (d, *J =* 22.1 Hz), 125.32, 127.55 (d, *J =* 2.9 Hz), 127.64, 129.37 (d, *J =* 8.5 Hz), 129.92, 140.12, 162.55, 164.52. HRMS (ESI): *m*/*z* = calcd. 396.95459 (M^+^H^+^, C_14_ H_9_ N_3_ F S I), found 397.96214, diff. 0.678 ppm.

**2-(2-Bromophenylamino)-5-(4-fluorophenyl)-1,3,4-thiadiazole (B2)**

Yield 32%, m.p. 166–168 °C. Spectral data were as follows: IR (cm^−1^) KBr: 3199 (NH), 2993 (CH aroma.), 1602 (C=N), 1578 (CH arom.), 741 (C-S). ^1^H NMR (DMSO-d_6_) δ 7.09 (td, *J =* 7.7, 1.6 Hz, 1H, ArH), 7.32–7.39 (m, 2H, ArH), 7.40–7.51 (m, 1H, ArH), 7.69 (dd, *J =* 8.1, 1.5 Hz, 1H, ArH), 7.82–7.99 (m, 2H, ArH), 8.11 (s, 1H, ArH), 9.89 (s, 1H, NH). ^13^C NMR (DMSO-d_6_) δ 115.56, 116.87 (d, *J =* 22.2 Hz), 124.04, 126.24, 127.43 (d, *J =* 3.0 Hz), 129.12, 129.49 (d, *J =* 8.5 Hz), 133.66, 163.61 (d, *J =* 248.3 Hz), 165.98. HRMS (ESI): *m*/*z* = calcd. 348.96846 (M^+^H^+^, C_14_ H_9_ N_3_ F S Br), found 349.97618, diff. 1.270 ppm.

**2-(2-Chlorophenylamino)-5-(4-fluorophenyl)-1,3,4-thiadiazole (B3)**

Yield 37%, m.p. 150–152 °C. Spectral data were as follows: IR (cm^−1^) KBr: 2995 (NH), 2912 (CH aroma.), 1594 (C=N), 1541 (CH arom.), 758 (C-S). ^1^H NMR (DMSO-d_6_) δ 7.13 (td, *J =* 7.7, 1.5 Hz, 1H, ArH), 7.34–7.42 (m, 3H, ArH), 7.53 (dd, *J =* 8.0, 1.5 Hz, 1H, ArH), 7.91–7.93 (m, 2H, ArH), 8.30 (s, 1H, ArH), 10.02 (s, 1H, NH). ^13^C NMR (DMSO-d_6_) δ 116.89 (d, *J =* 22.1 Hz), 122.63, 123.80, 125.07, 127.43 (d, *J =* 3.1 Hz), 128.50, 129.56 (d, *J =* 8.8 Hz), 130.30, 163.67 (d, *J =* 244 Hz), 165.43. HRMS (ESI): *m*/*z* = calcd. 305.01897 (M^+^H^+^, C_14_ H_9_ N_3_ F S Cl), found 306.02604, diff. −0.689 ppm.

### 3.2. Cell Lines

The experiments were conducted using MDA-MB-231 and MCF-7 human breast cancer cell lines, as well as human fibroblasts (HMF). All cell lines were sourced from the American Type Culture Collection (ATCC; Manassas, VA, USA). Cells were maintained under standard culture conditions (37 °C, 5% CO_2_). Breast cancer cells were grown in Dulbecco’s Modified Eagle Medium (DMEM) supplemented with 10% fetal bovine serum (FBS), while fibroblasts were cultured in Eagle’s Minimum Essential Medium (EMEM) with the same serum concentration. To prevent microbial contamination, penicillin (100 U/mL) and streptomycin (100 µg/mL) were added to the media. All cell culture reagents were obtained from Sigma Aldrich (St. Louis, MO, USA).

### 3.3. MTT Assay

Cell viability was evaluated using the MTT assay kit (Sigma Aldrich, St. Louis, MO, USA). On day one of the experiment, MCF-7, MDA-MB-231 cells and human fibroblasts were seeded into 96-well culture plates (Nunc, Roskilde, Denmark) at a density of 1 × 10^5^ cells/mL (human fibroblast cell line) and 5 × 10^4^ cells/mL (MCF-7, MDA-MB-231 cell lines). After 24 h of incubation, the culture medium was removed and replaced with serial dilutions of test compounds or anastrozole (1, 12.5, 25, 50, 100 µM). Control wells received fresh medium only. Cells were incubated for another 24 h under standard conditions. Following this, 15 µL of MTT solution (5 mg/mL) was added to each well and incubated for 3 h. Afterward, a 10% sodium dodecyl sulfate (SDS) solution was added, and the plates were left overnight. On the following day, absorbance was measured at 570 nm using a microplate reader (Epoch, BioTek Instruments, Inc., Winooski, VT, USA) with Gen5 software (version 2.01, BioTek Instruments).

### 3.4. Statistical Analysis

Data were analyzed using GraphPad software version 5.01 (GraphPad Software, Inc., La Jolla, CA, USA). Results are expressed as means ± standard deviation (±SD). IC_50_ values were calculated using the IC_50_ calculator provided by AAT Bioquest [17].

### 3.5. Molecular Docking

The crystal structure of human cytochrome P450 aromatase co-crystallized with a novel C6-substituted androsta-1,4-diene-3,17-dione inhibitor (PDB ID: 4GL7) was retrieved from the Protein Data Bank [18]. The Protein Preparation Wizard was used to assign bond orders, determine appropriate ionization states of amino acid residues, and identify steric clashes. A receptor grid was generated using the OPLS5 force field [19], centering a 12 Å grid box on the co-crystallized inhibitor. Three-dimensional ligand structures were prepared with LigPrep v3.7, and ionization states at pH 7.4 ± 1.0 were assigned using Epik v3.5. Automated flexible docking was performed with Glide at the SP precision level [20], generating ten poses per ligand. The docking procedure was validated by re-docking the native inhibitor into the active site of the enzyme, yielding a predicted pose with an RMSD below 1 Å relative to the crystallographic conformation (best-scored pose).

### 3.6. Molecular Dynamics Simulations

All-atom molecular dynamics (MD) simulations were performed using Desmond (Schrödinger Software, Schrodinger, LLC: New York, NY, USA) for a total duration of 200 ns. Each ligand–receptor complex was solvated in an explicit TIP4P water box, and the OPLS5 force field was applied to all atoms [19]. To reproduce physiological conditions, NaCl ions were added to achieve a final ionic strength of 0.15 M. Simulations were conducted with a 2 fs integration time step, and trajectory frames were saved every 100 ps. An NPT ensemble was employed, with temperature controlled using the Nose–Hoover chain thermostat (relaxation time: 1.0 ps) and pressure maintained by the Martyna–Tobias–Klein barostat (relaxation time: 1.0 ps).

## 4. Conclusions

In the search for new anticancer agents, six novel 1,3,4-thiadiazole derivatives containing two halogen-substituted aromatic rings in their structure were designed and synthesized. The conducted studies demonstrated that against the MCF-7 cell line all compounds exhibited concentration-dependent cytotoxic activity. The strongest anticancer effect was observed for compound **A2** (IC_50_ = 52.35 µM), as well as for compounds **A3** (IC_50_ = 54.81 µM), **B1** (IC_50_ = 53.9 µM), and **B3** (IC_50_ = 54.1 µM), indicating their comparable efficacy against this cell line. In contrast, in the case of the MDA-MB-231 cell line, none of the tested compounds, similar to anastrozole used as a reference drug, achieved an IC_50_ value below 100 µM, indicating low cytotoxic activity against estrogen-independent cancer cells. The obtained results suggest a mechanism of activity related to aromatase inhibition. Compound **B3** was subjected to in silico analyses due to its highest potential for therapeutic application. It was shown that, in complex with aromatase, the compound occupies nearly the same binding site as the native co-crystallized inhibitor. The interactions described for this most promising 1,3,4-thiadiazole derivative are consistent with those typically observed for potent aromatase inhibitors, suggesting that **B3** effectively reproduces the key molecular features required for efficient enzyme binding and inhibition.

All compounds from the studied series exhibited certain anticancer activity, the mechanism of which is likely associated with aromatase inhibition. A common structural feature of this group was a phenyl ring substituted with fluorine atoms in the *para* and *meta* positions. Therefore, it is worthwhile to continue research on this class of compounds by expanding the series to include derivatives containing fluorine in the *ortho* position as well as compounds bearing two fluorine-substituted phenyl rings. This may create an opportunity to develop more effective and less toxic aromatase inhibitors.

## Figures and Tables

**Table 1:** IC_50_ values of the tested compounds were determined based on concentration-response curve analysis. IC_50_ values were calculated using the IC_50_ Calculator (AAT Bioquest; https://www.aatbio.com/tools/ic50-calculator, accessed on 26 November 2025).

	IC_50_ [µM]						
Cell Line	A1	A2	A3	B1	B2	B3	Anastrozole
MDA-MB-231	>100	>100	>100	>100	>100	>100	>100
MCF-7	65.95	82.48	54.81	53.9	82.48	54.1	>100
HMF	51.01	41.8	36.9	45.58	41.8	>100	>100

## Data Availability

Data are contained within the article and Supplementary Materials.

## Supplementary Materials

The following supporting information can be downloaded at: https://www.mdpi.com/article/10.3390/molecules30244744/s1, ^1^H NMR spectra, ^13^C NMR spectra, IR spectra, MS spectra, Molecular Dynamics Analysis of Binding Modes of Compound **B3.**
