# Supplementary material for: Synthesis and In Vitro Evaluation of Anticancer Activity of Fluorophenyl Derivatives of 1,3,4-Thiadiazole Against Estrogen-Dependent Breast Cancer
Source: Molecules. 2025 Dec 12;30(24):4744. doi: 10.3390/molecules30244744 (PMC12736086; doi:10.3390/molecules30244744)
Supplement: Supplementary file 1 [file molecules-30-04744-s001.zip › molecules-4003356-supplementary.pdf]

Supplementary Information

Synthesis and In Vitro Evaluation of Anticancer Activity of Fluorophenyl Derivatives of 1,3,4-Thiadiazole Against Estrogen-Dependent Breast Cancer

Sara Janowska[1], Anna Makuch-Kocka[2], Rafał Kurczab[3], Oleg M. Demchuk[4] and Monika Wujec[5] *

[1] Department of Experimental and Clinical Pharmacology, Medical University of Lublin, 4A Chodźki Street, 20-093 Lublin, Poland, sarajanowska@umlub.pl
[2] Department of Pharmacology, Medical University of Lublin, 4A Chodźki Street, 20-093 Lublin, Poland
[3] Department of Medicinal Chemistry, Maj Institute of Pharmacology, Polish Academy of Sciences, Smetna 12, 31-343 Kraków, Poland
[4] Faculty of Medicine, The John Paul II Catholic University of Lublin, 1J Konstantynów Street, 20-708 Lublin, Poland
[5] Department of Organic Chemistry, Medical University of Lublin, 4A Chodźki Street, 20-093 Lublin, Poland, monikawujec@umlub.pl

*Correspondence: monika.wujec@umlub.pl (M.W.) and sara.janowska@umlub.pl (S.J.)

Table of contents:

^{1}H NMR spectra of thiosemicarbazide derivatives ……………………………………………………………………………………………………….2
^{1}H NMR spectra of 1,3,4-thiadiazole derivatives ……………………………………………………………………………………………………….8
^{13}C NMR spectra of thiosemicarbazide derivatives……………………………………………………………………………………………………….14
^{13}C NMR spectra of 1,2,4-thiadiazole derivatives……………………………………………………………………………………………………….20
IR spectra of 1,2,4-thiadiazole derivatives……………………………………………………………………………………………………….26
MS spectra of 1,2,4-thiadiazole derivatives……………………………………………………………………………………………………….32
Molecular Dynamics Analysis of Binding Modes of Compound B3……………………………………………………………………………..38

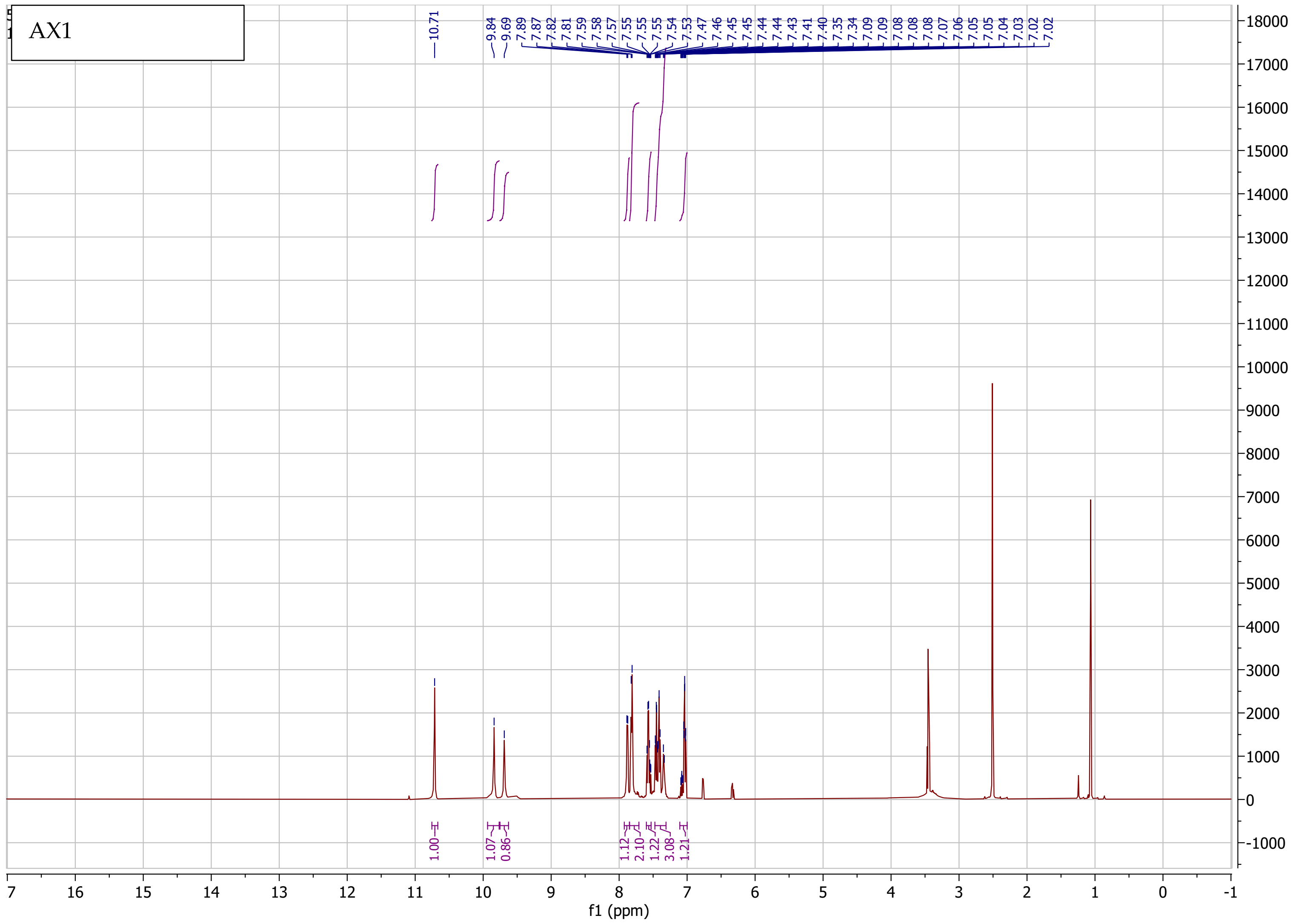

Figure S1. The ^{1}H NMR of compound AX1.

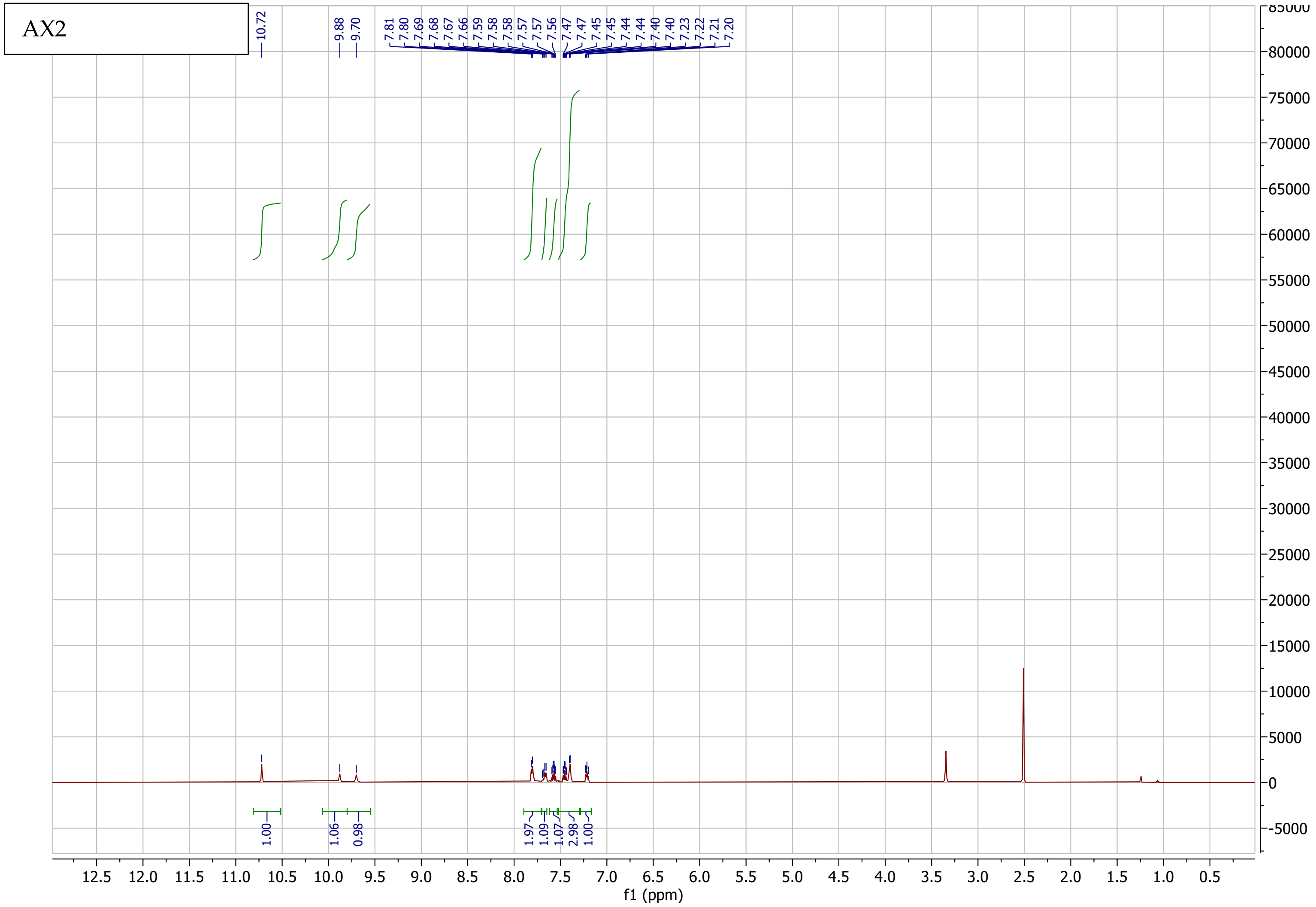

Figure S2. The ^{1}H NMR of compound AX2.

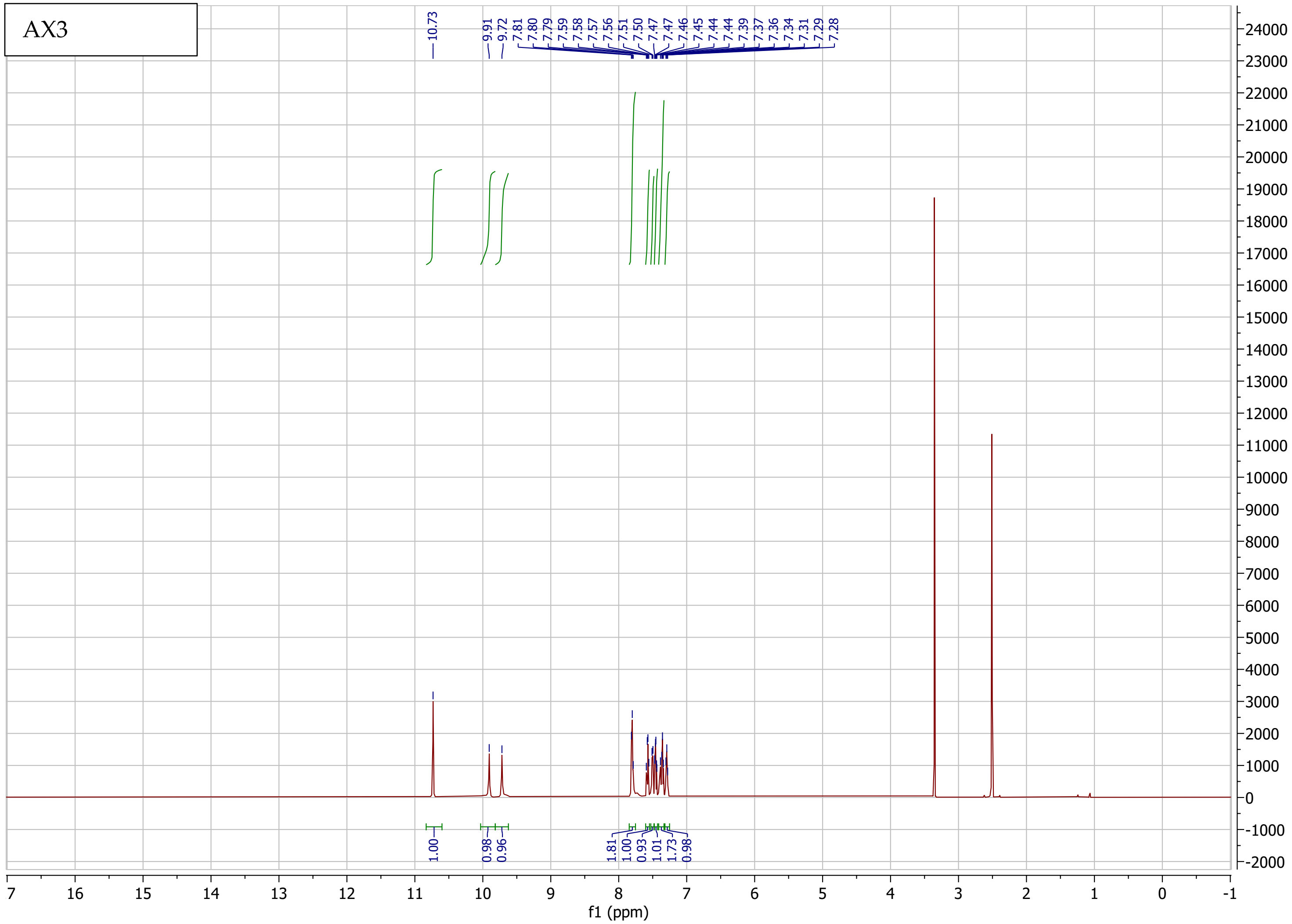

Figure S3. The ^{1}H NMR of compound AX3.

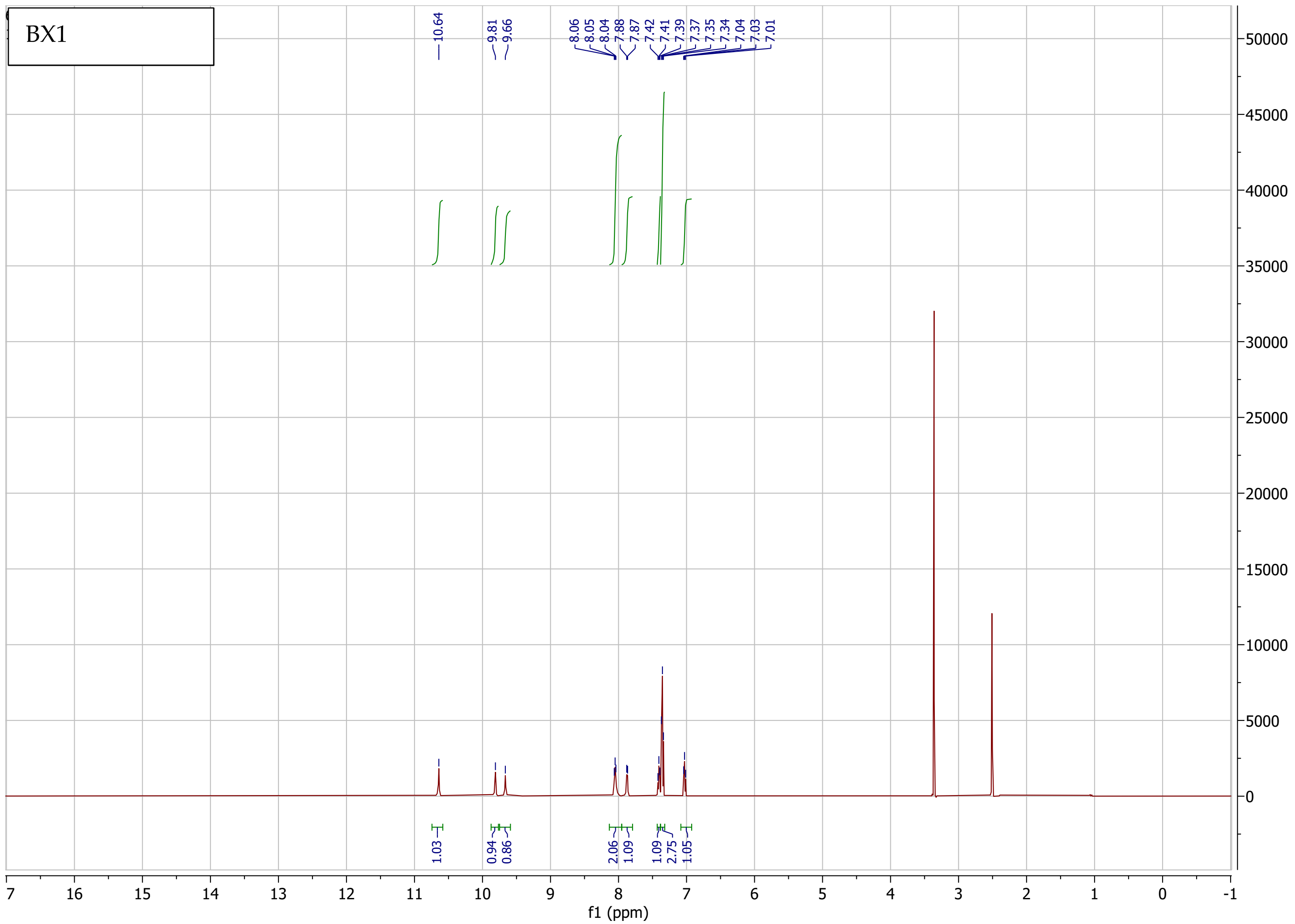

Figure S4. The ^{1}H NMR of compound BX1.

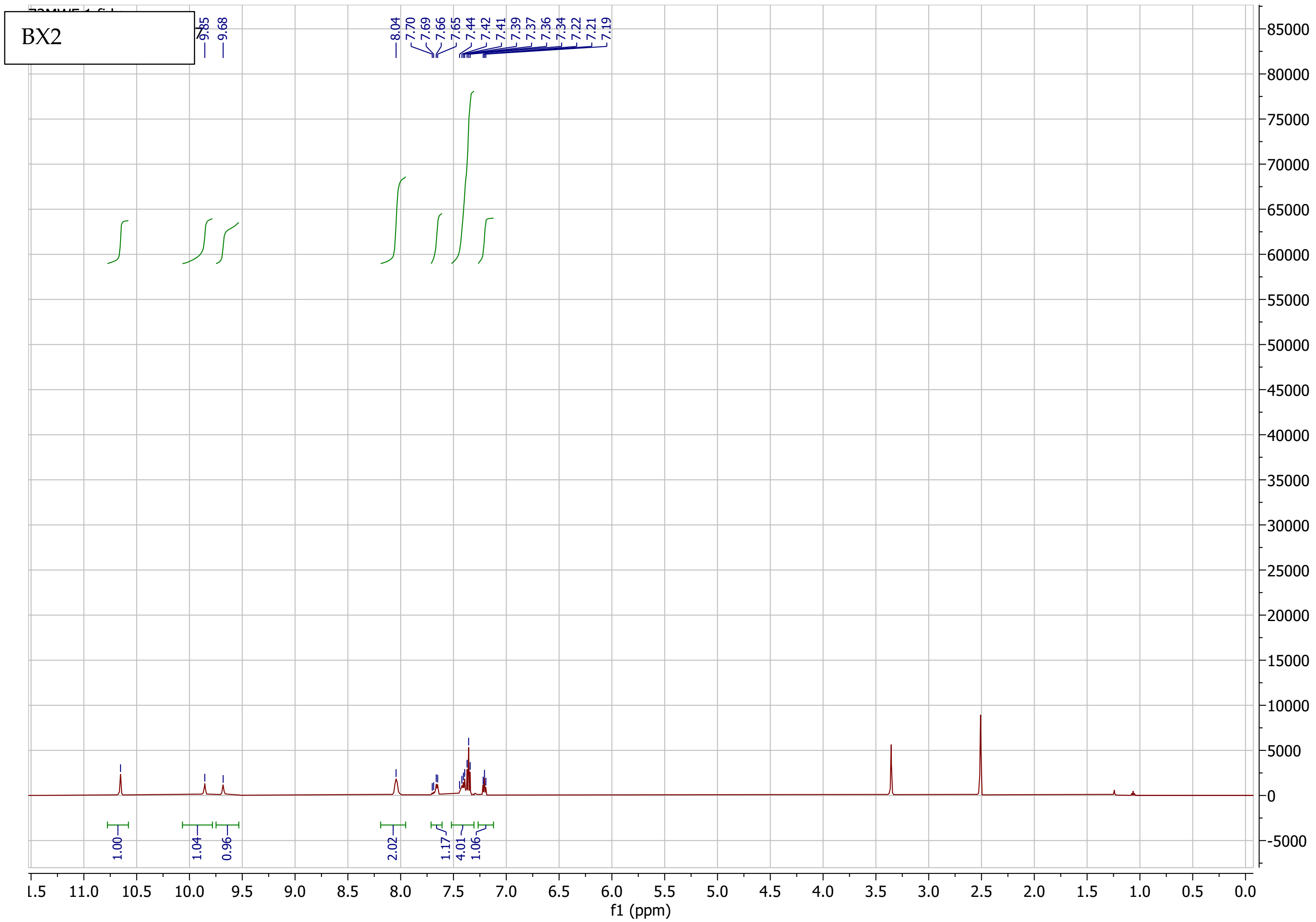

Figure S5. The ^{1}H NMR of compound BX2.

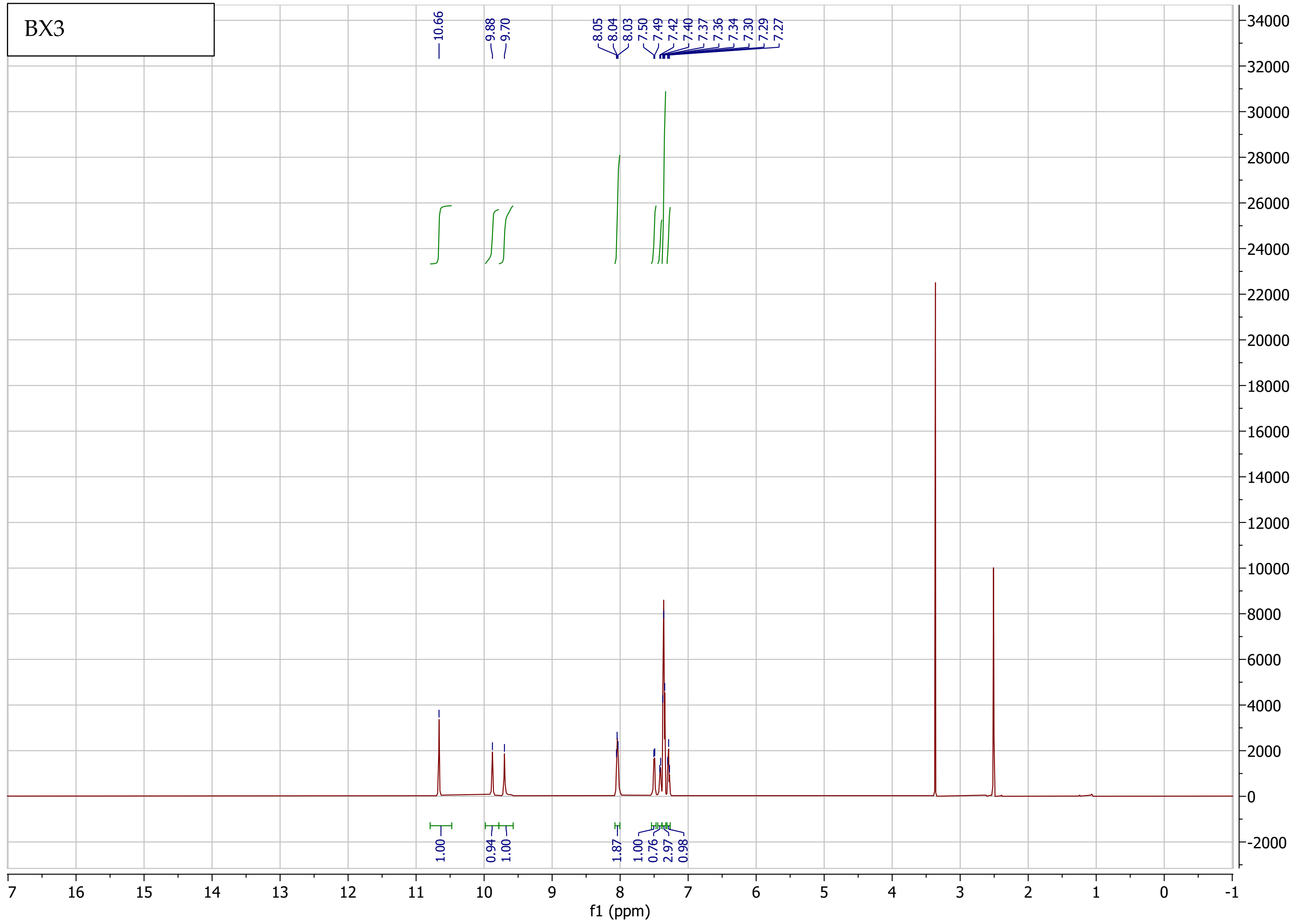

Figure S6. The ^1H NMR of compound BX3.

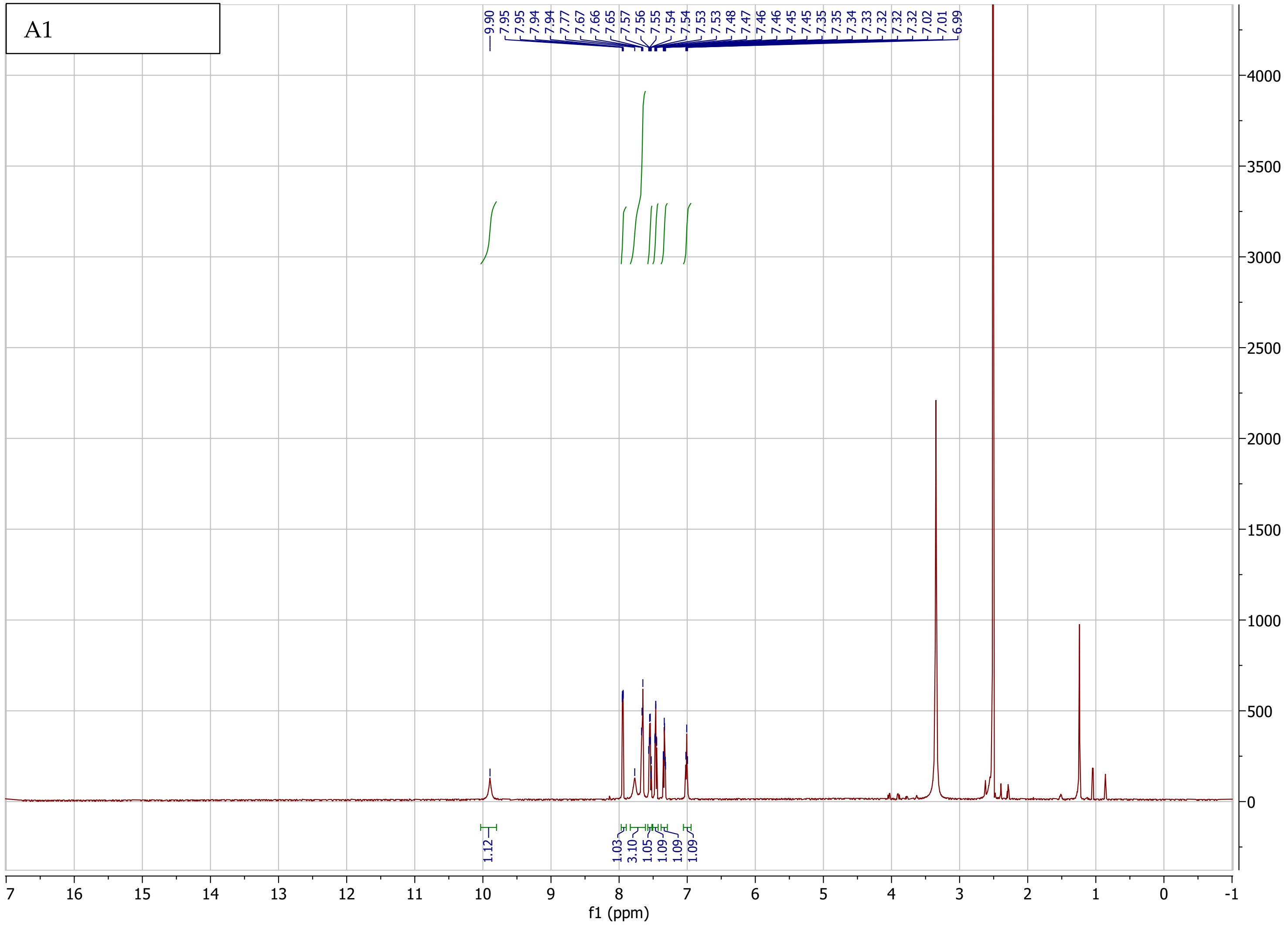

Figure S7. The ^{1}H NMR of compound A1.

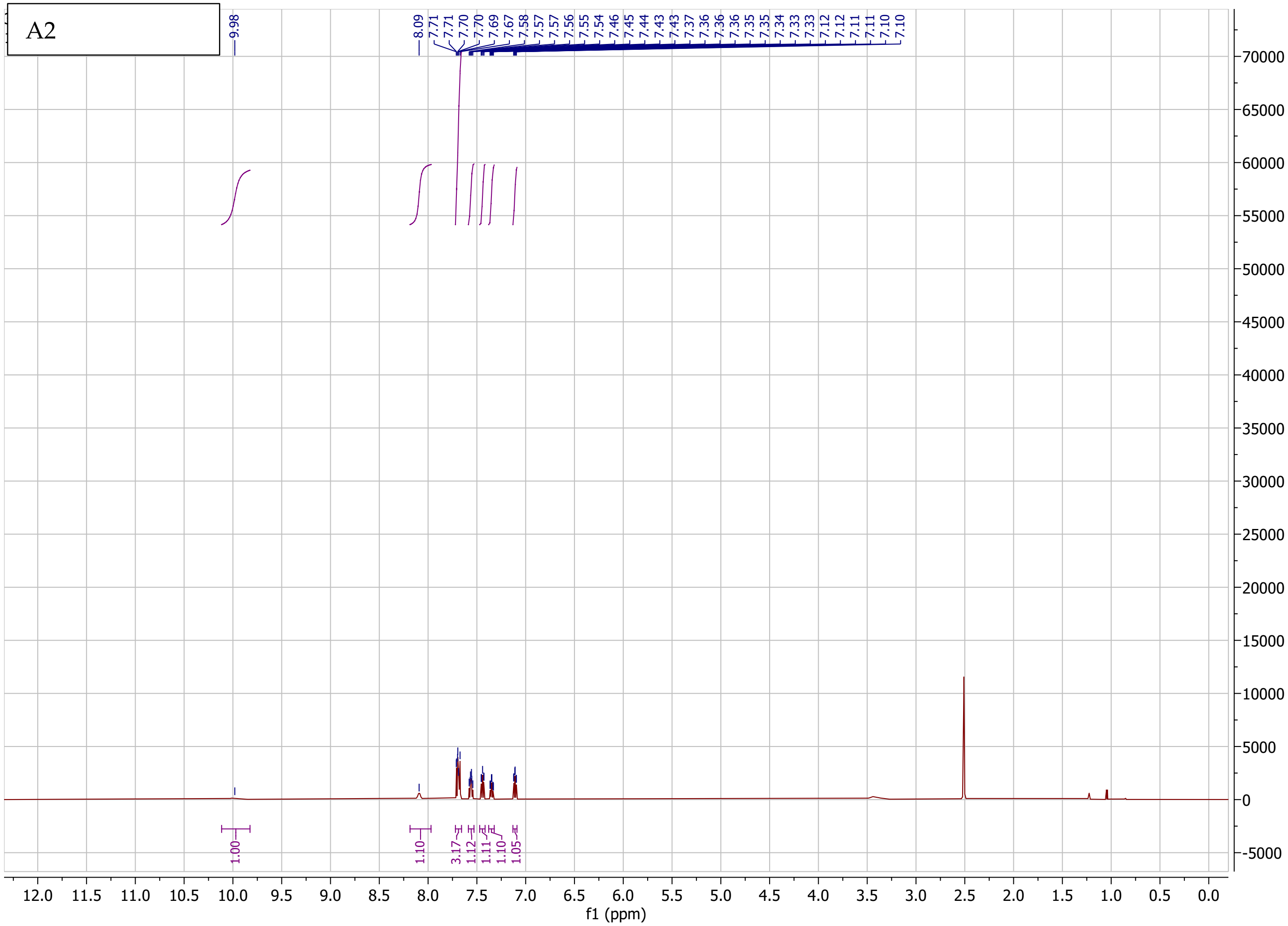

Figure S8. The ¹H NMR of compound A2

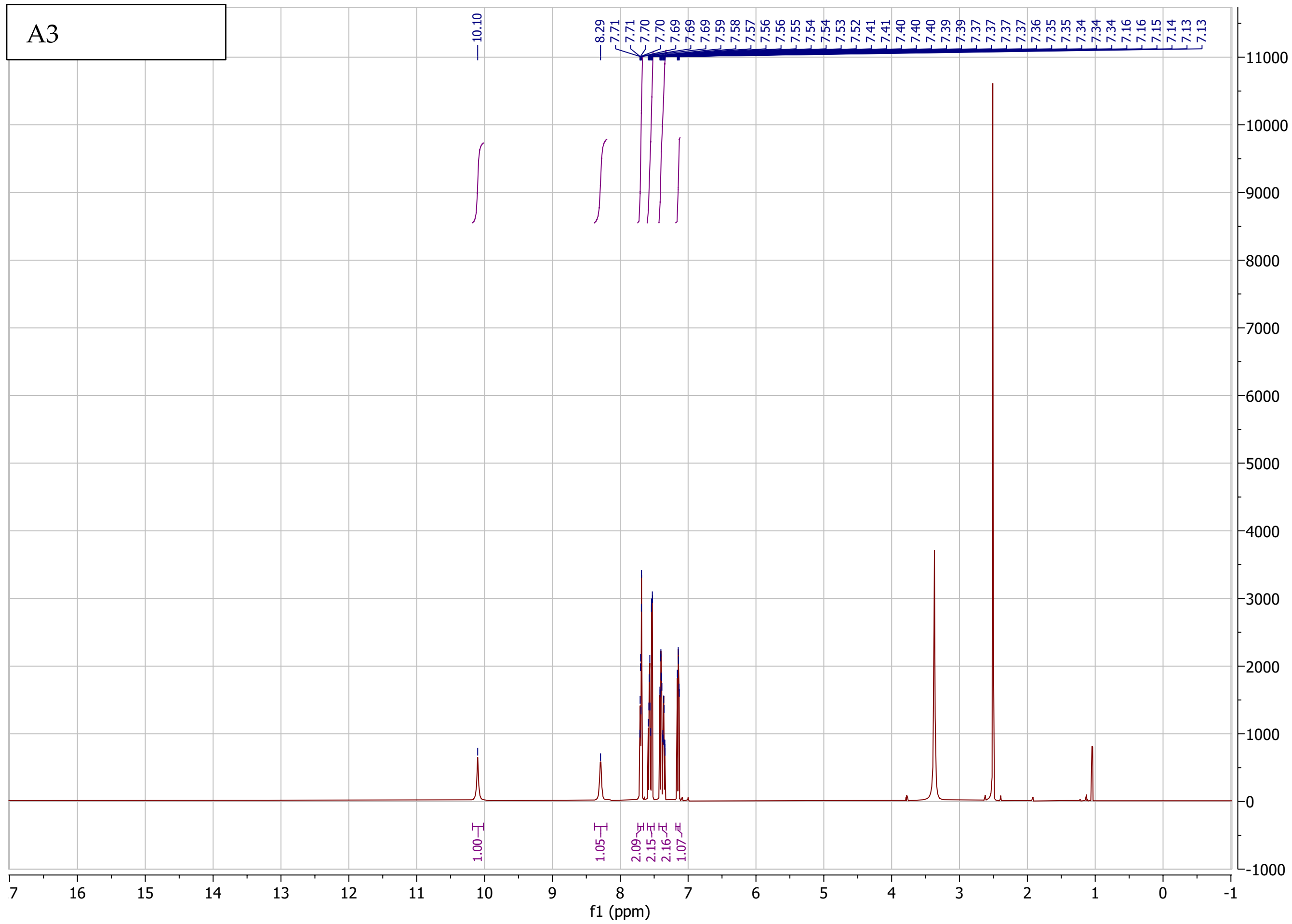

Figure S9. The ^{1}H NMR of compound A3

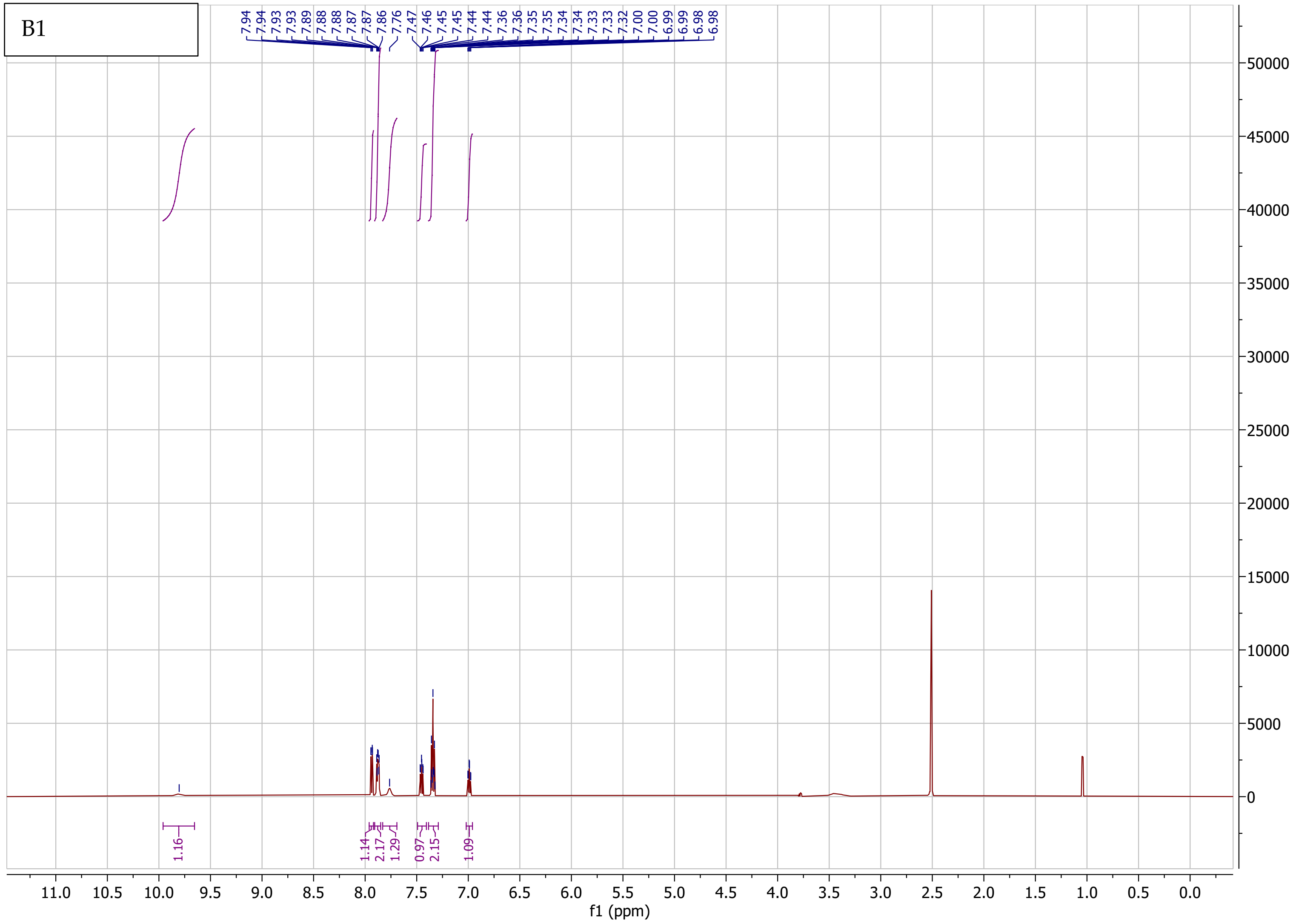

Figure S10. The ^{1}H NMR of compound B1

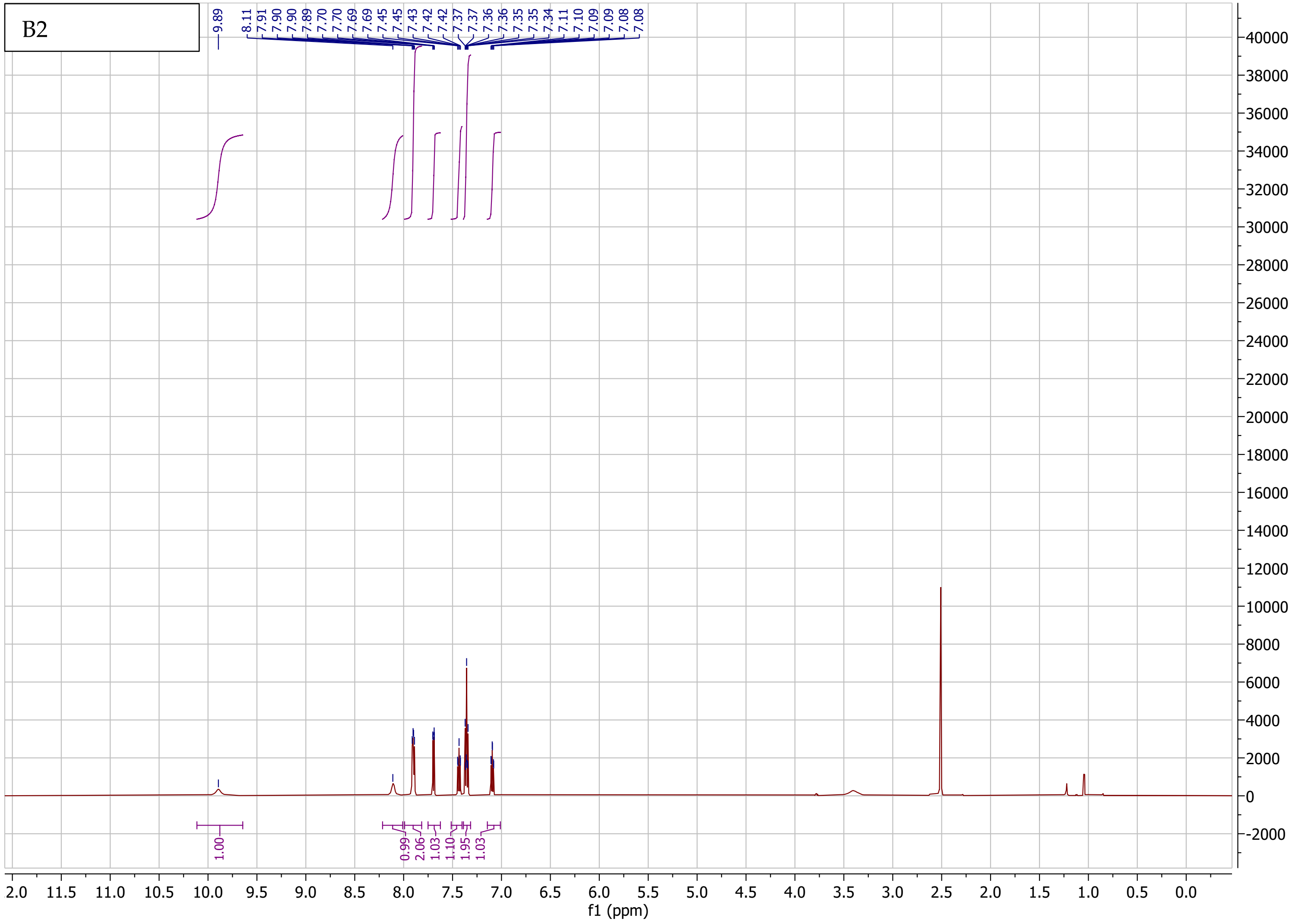

Figure S11. The ^{1}H NMR of compound B2.

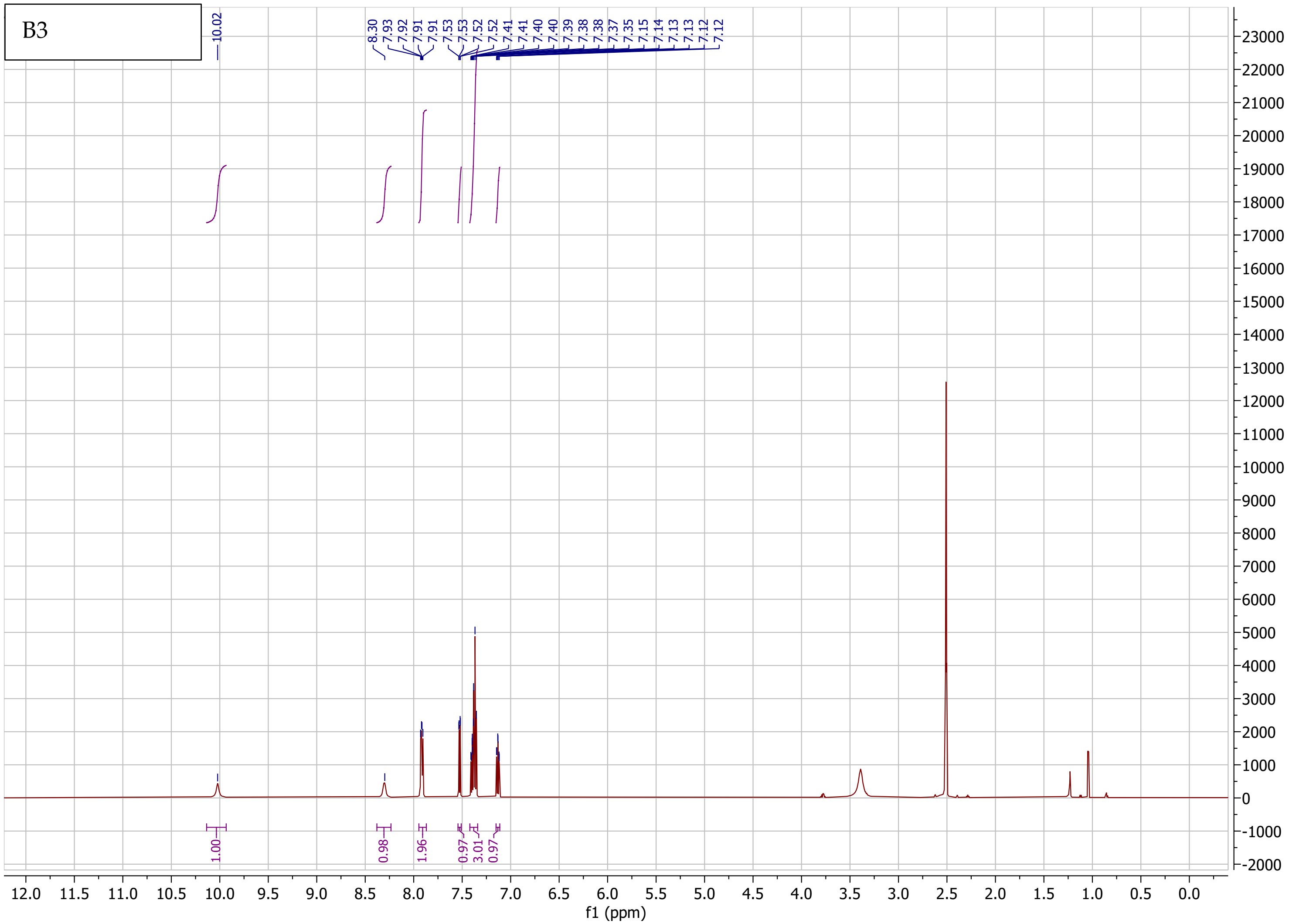

Figure S12. The ¹H NMR of compound B3.

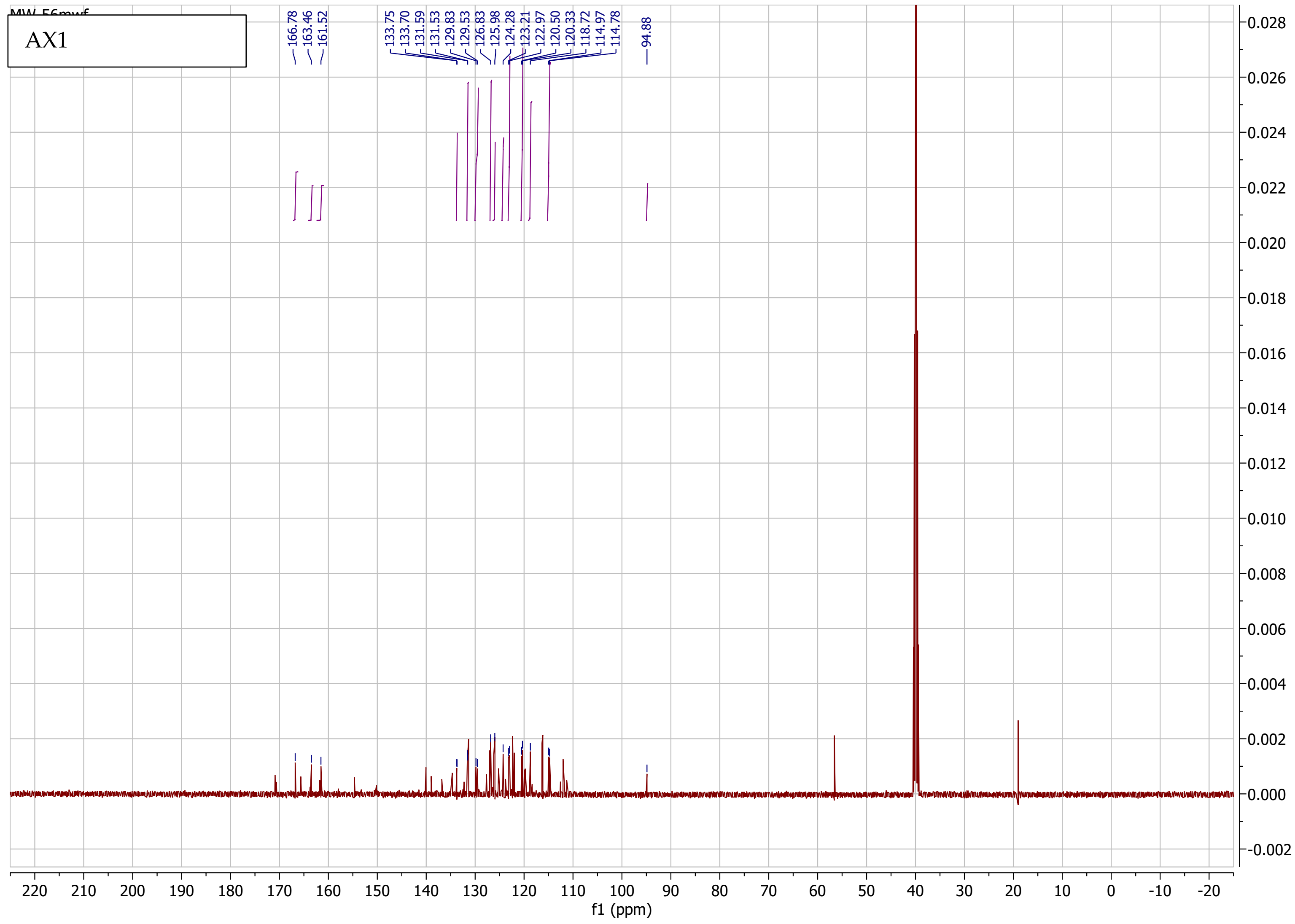

Figure S13. The ^{13}C NMR of compound AX1.

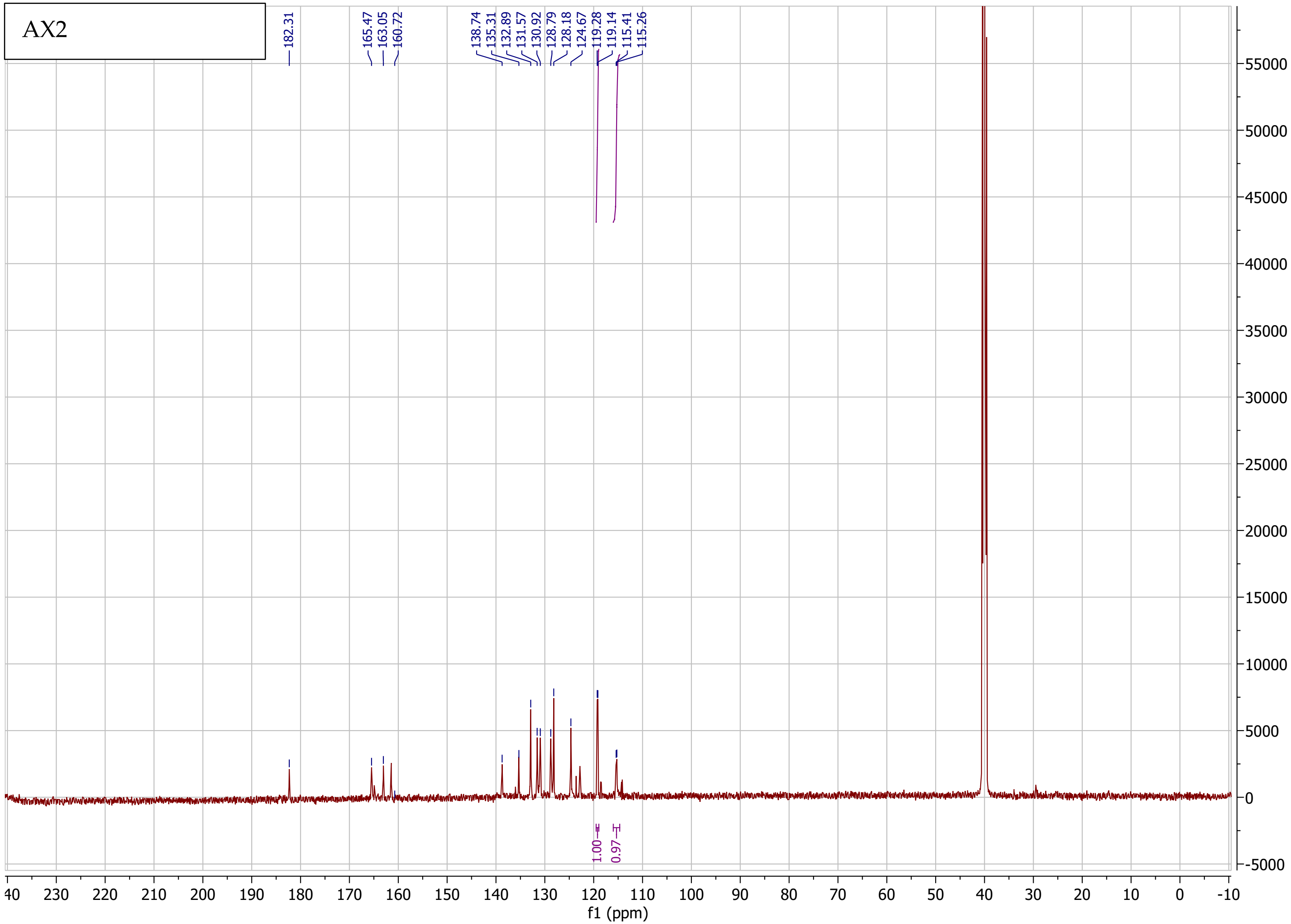

Figure S14. The ^{13}C NMR of compound AX2.

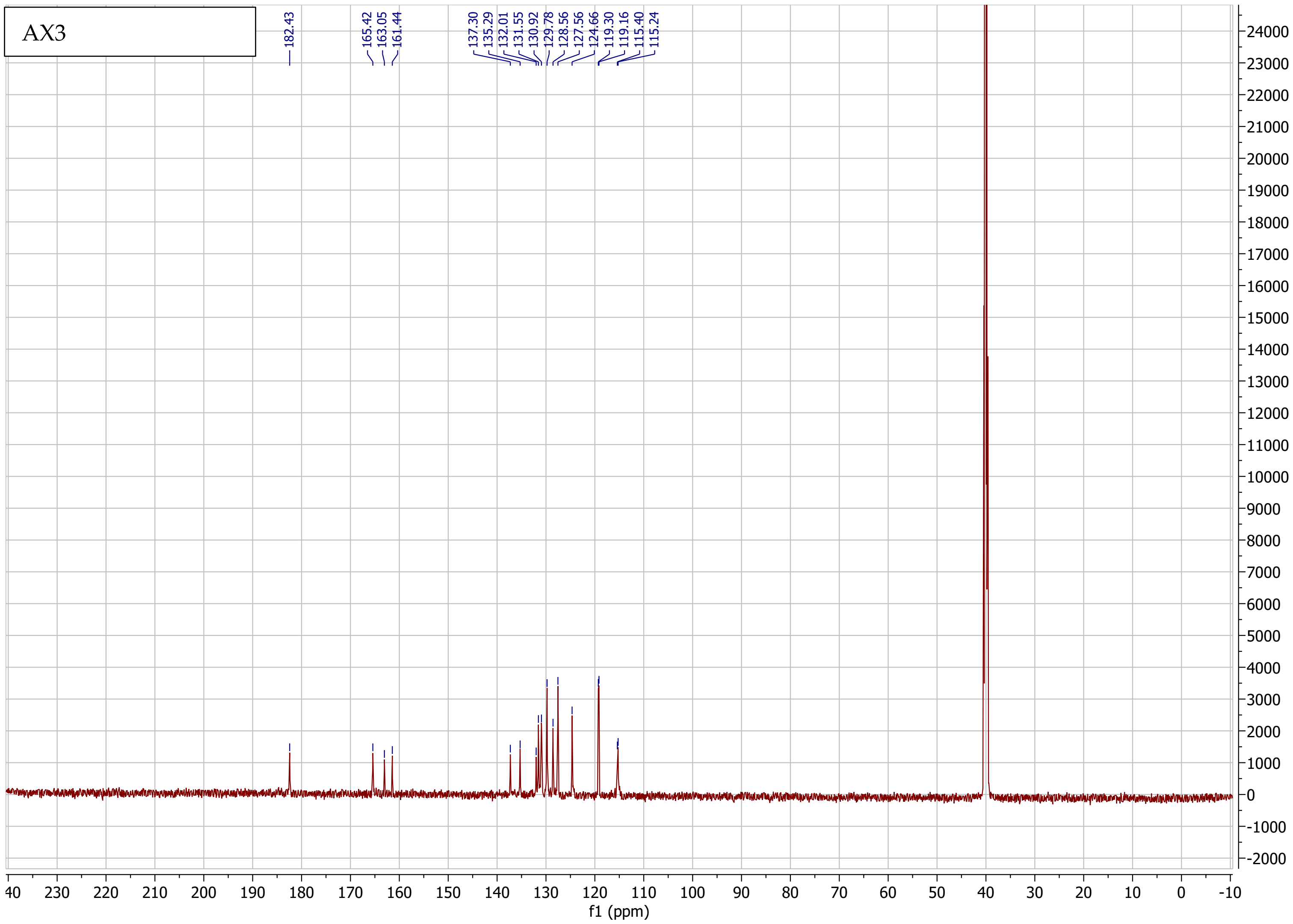

Figure S15. The ^{13}C NMR of compound AX3.

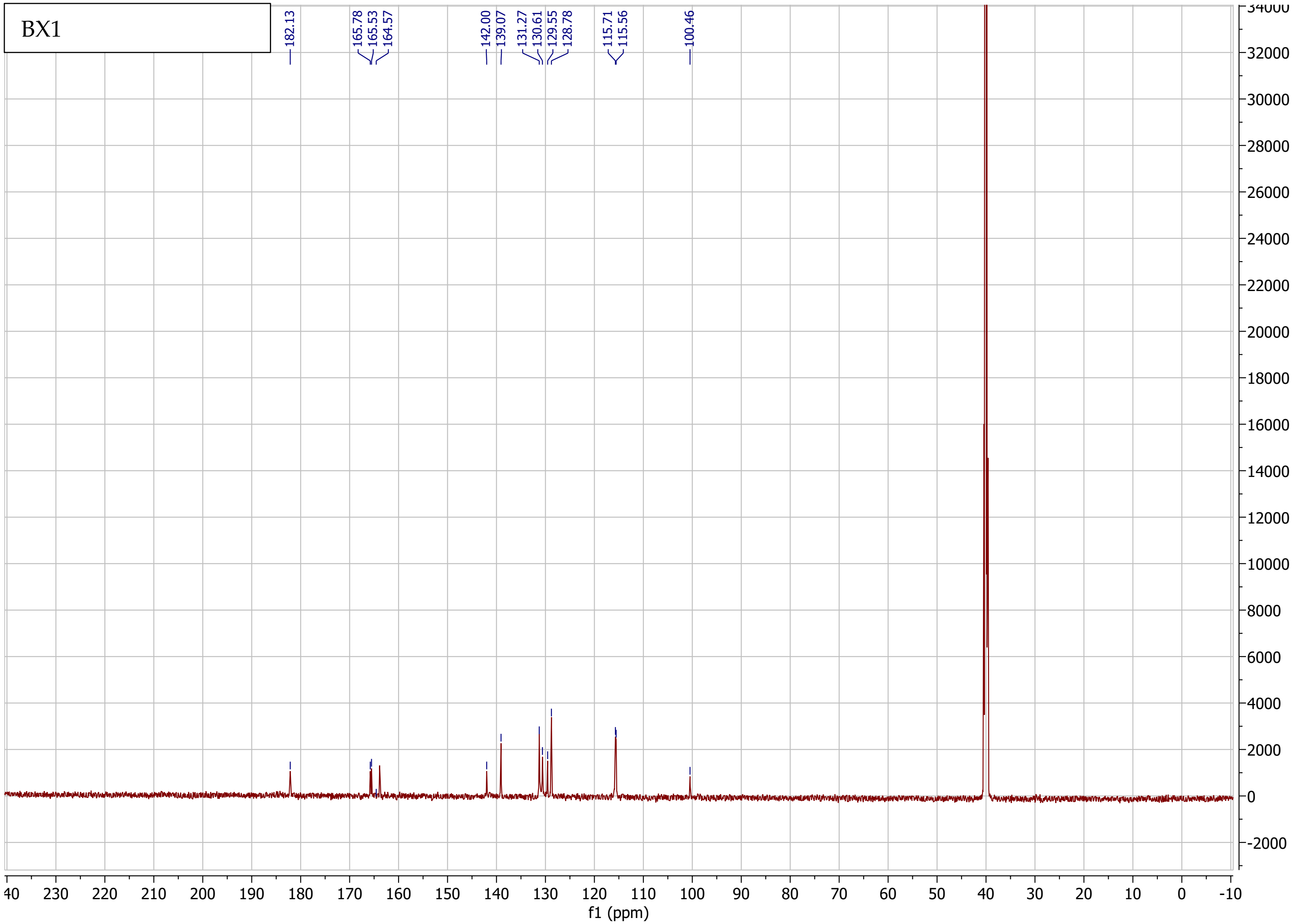

Figure S16. The ^{13}C NMR of compound BX1.

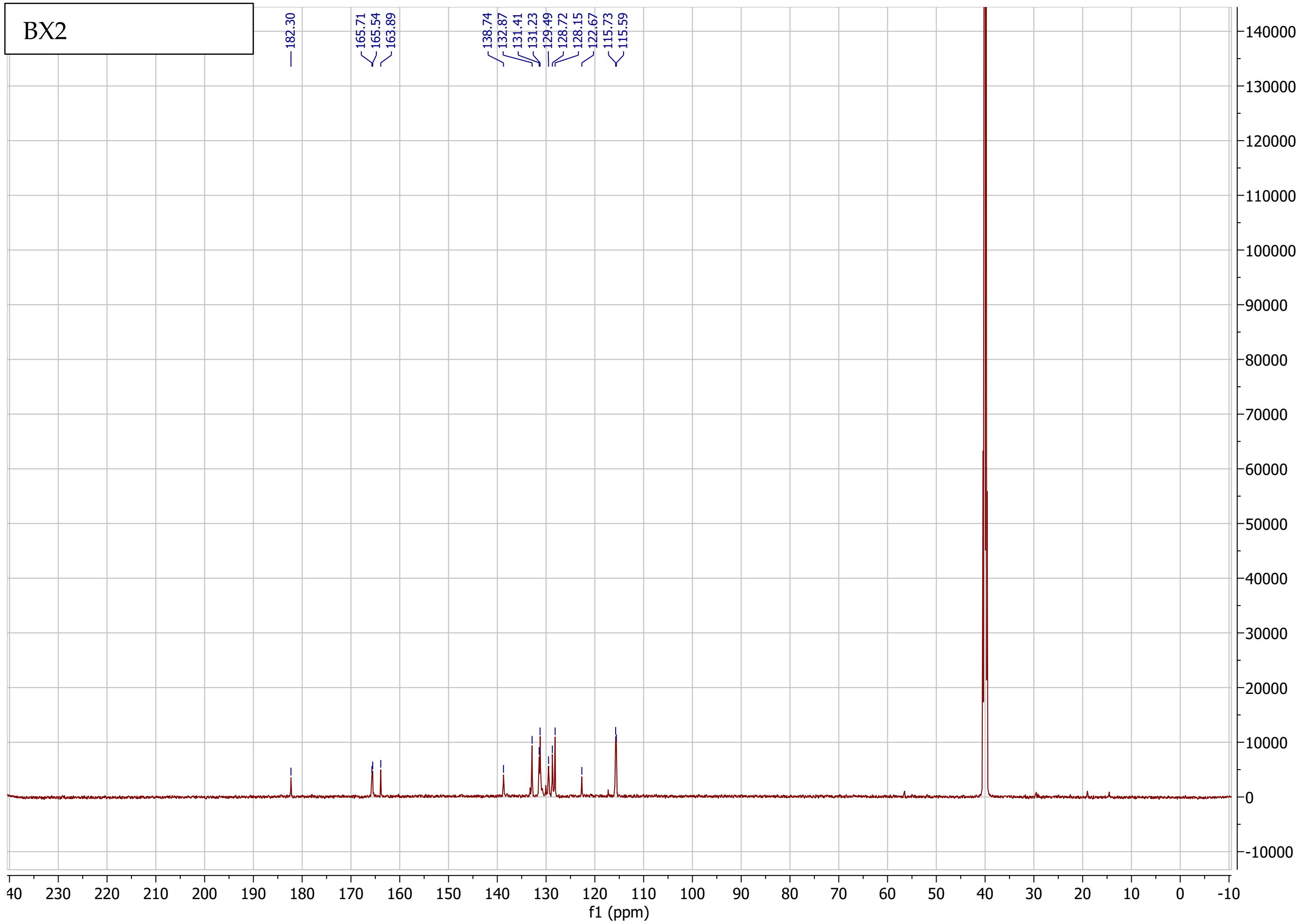

Figure S17. The ^{13}C NMR of compound BX2.

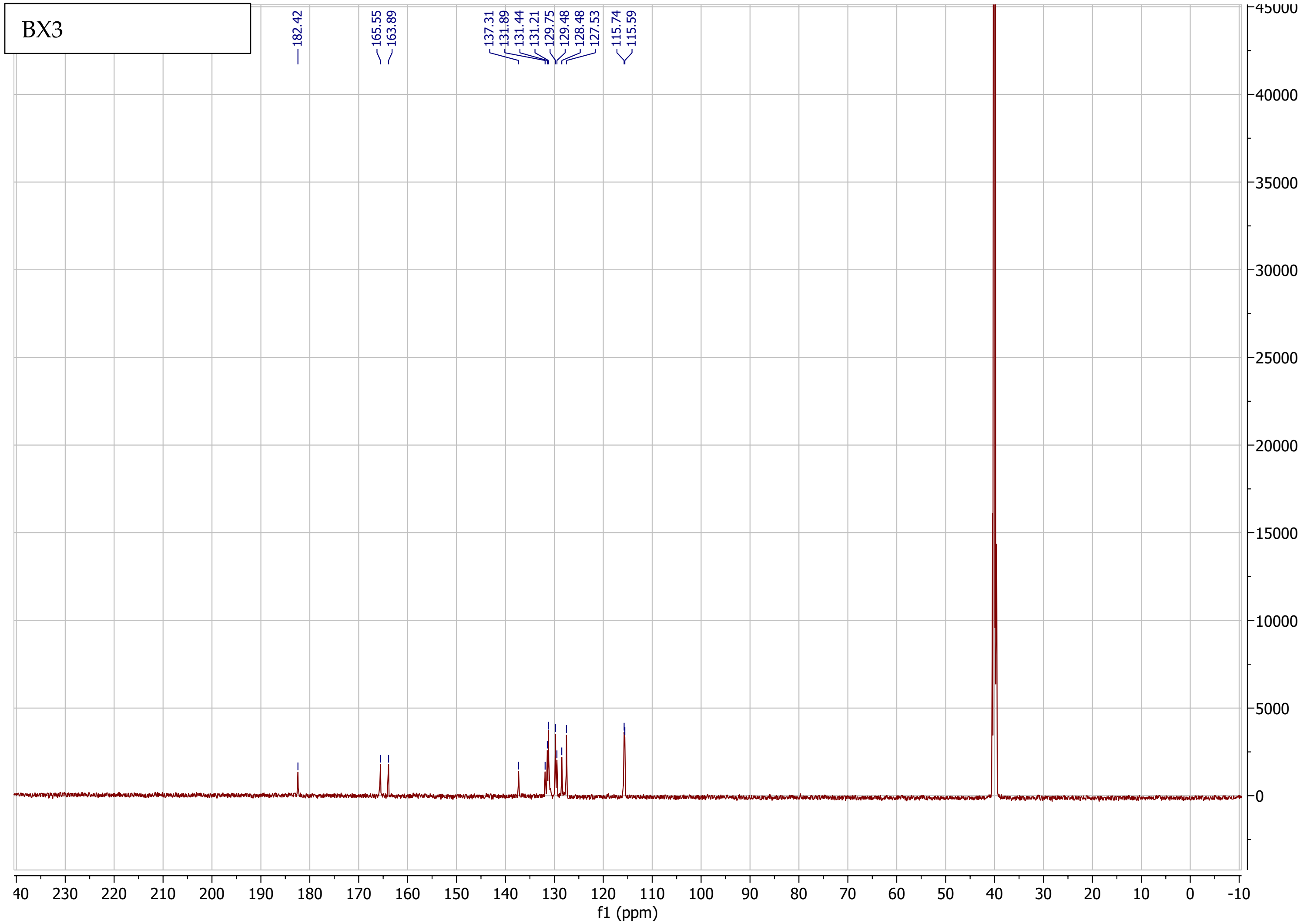

Figure S18. The ^{13}C NMR of compound BX3.

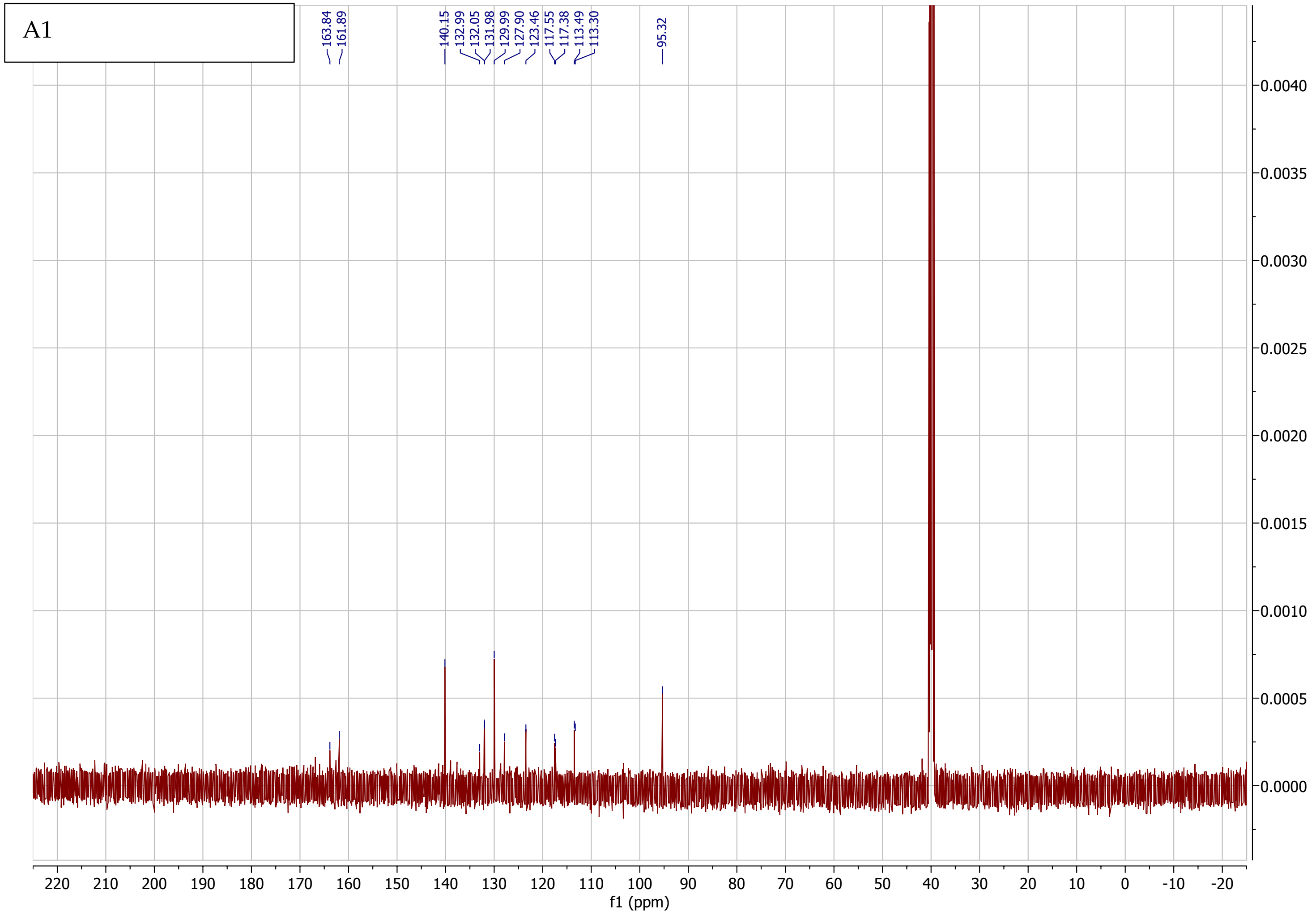

Figure S19. The ^{13}C NMR of compound A1.

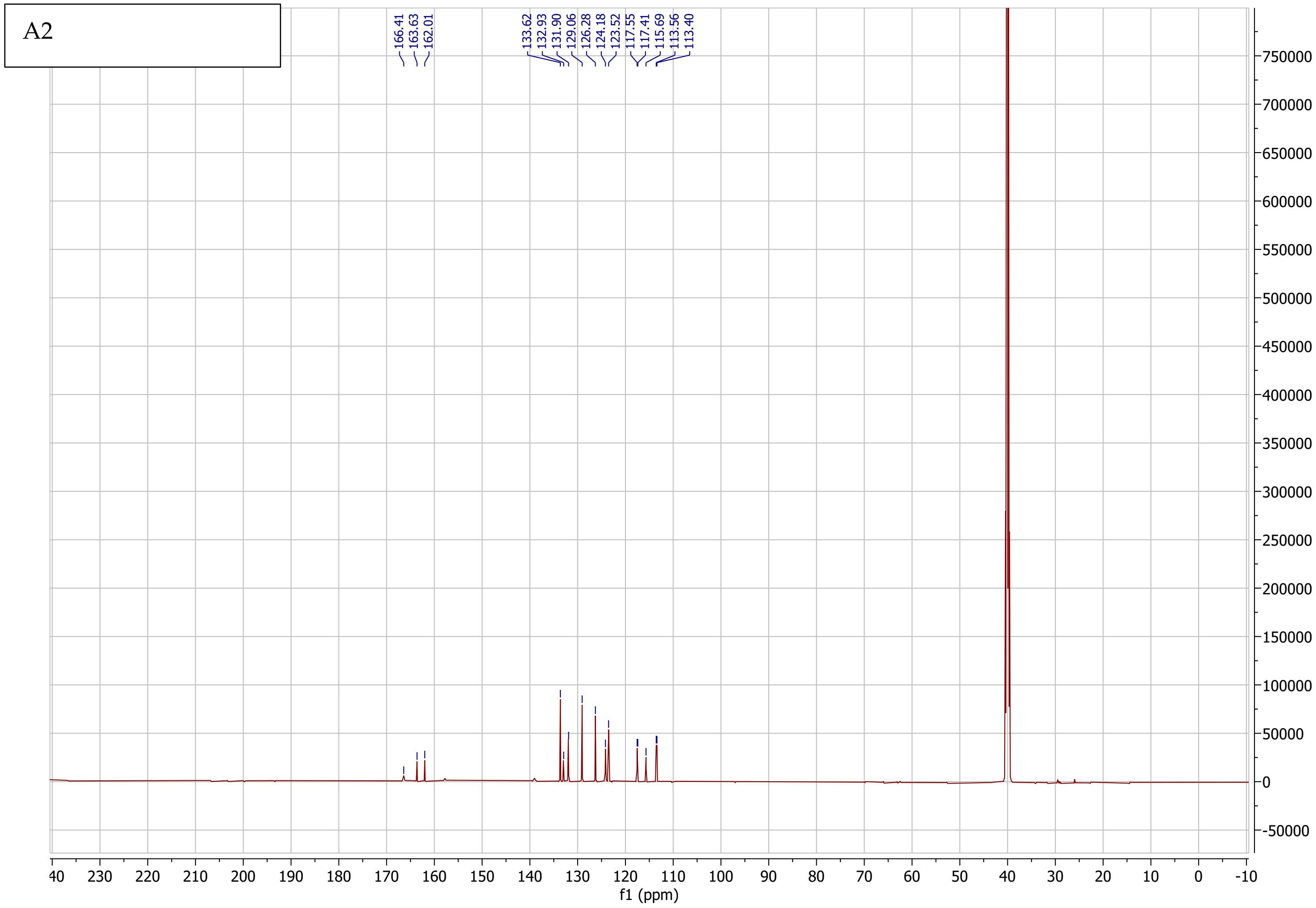

Figure S20. The ^{13}C NMR of compound A2.

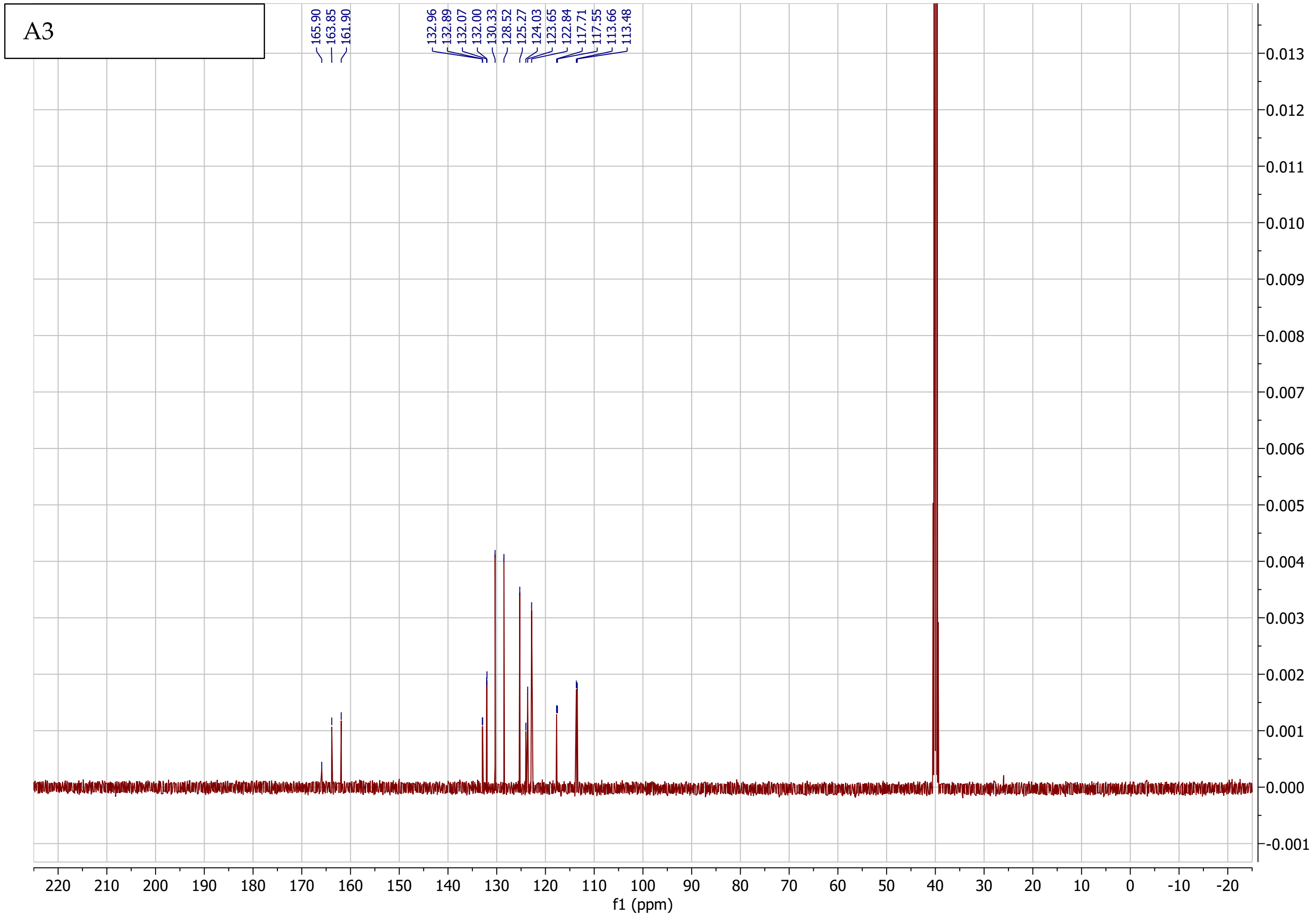

Figure S21. The ^{13}C NMR of compound A3.

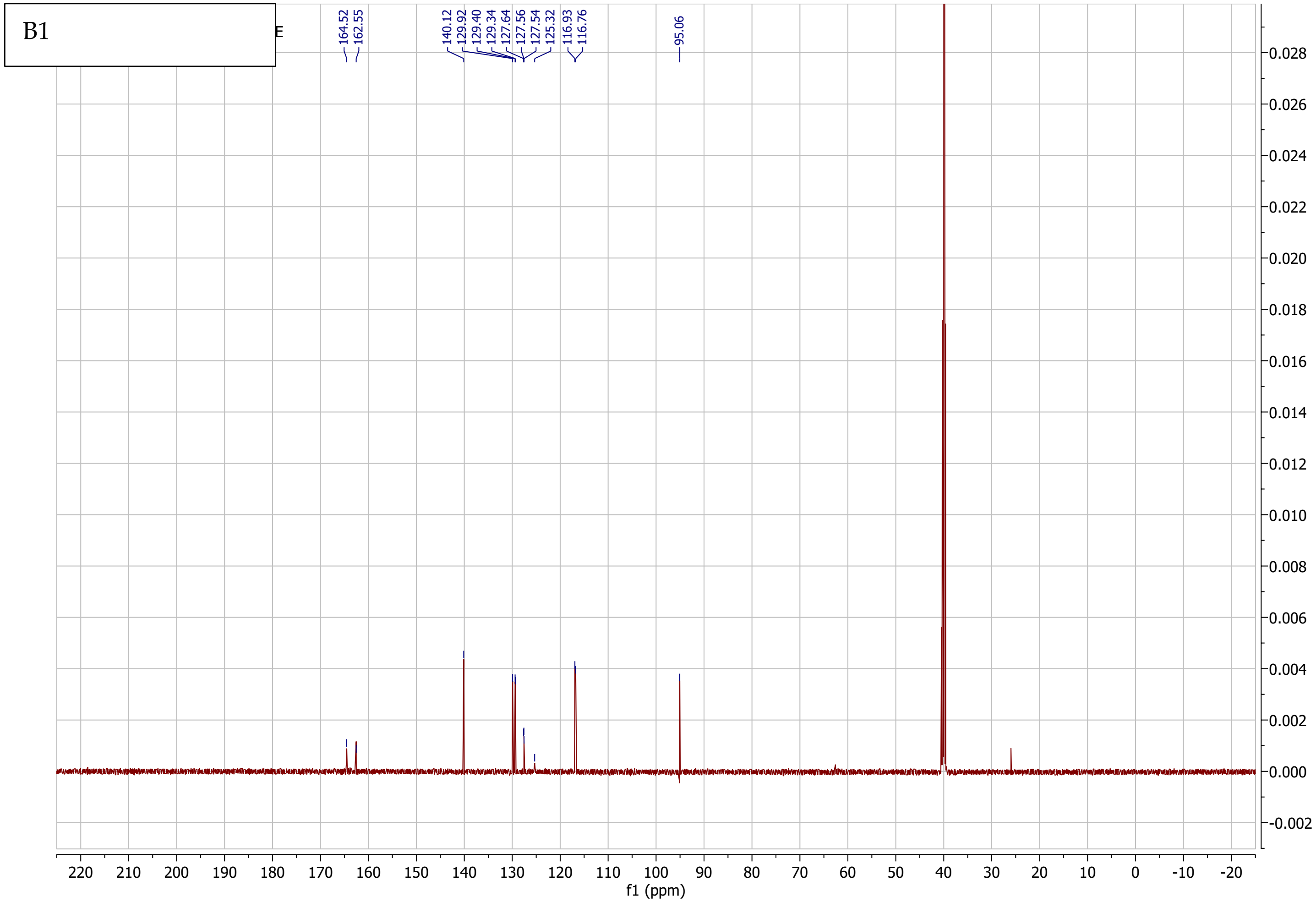

Figure S22. The ^{13}C NMR of compound B1.

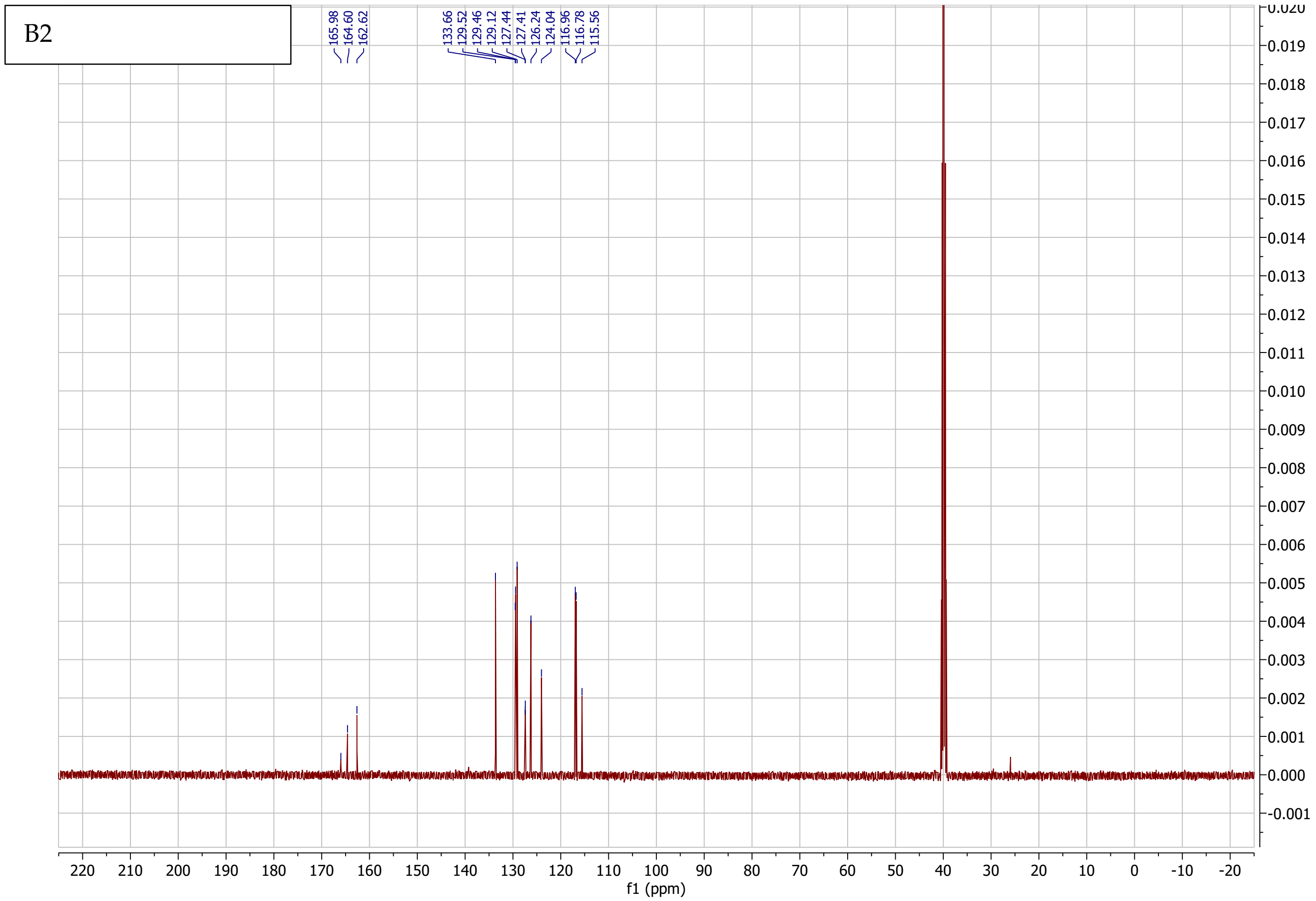

Figure S23. The ^{13}C NMR of compound B2.

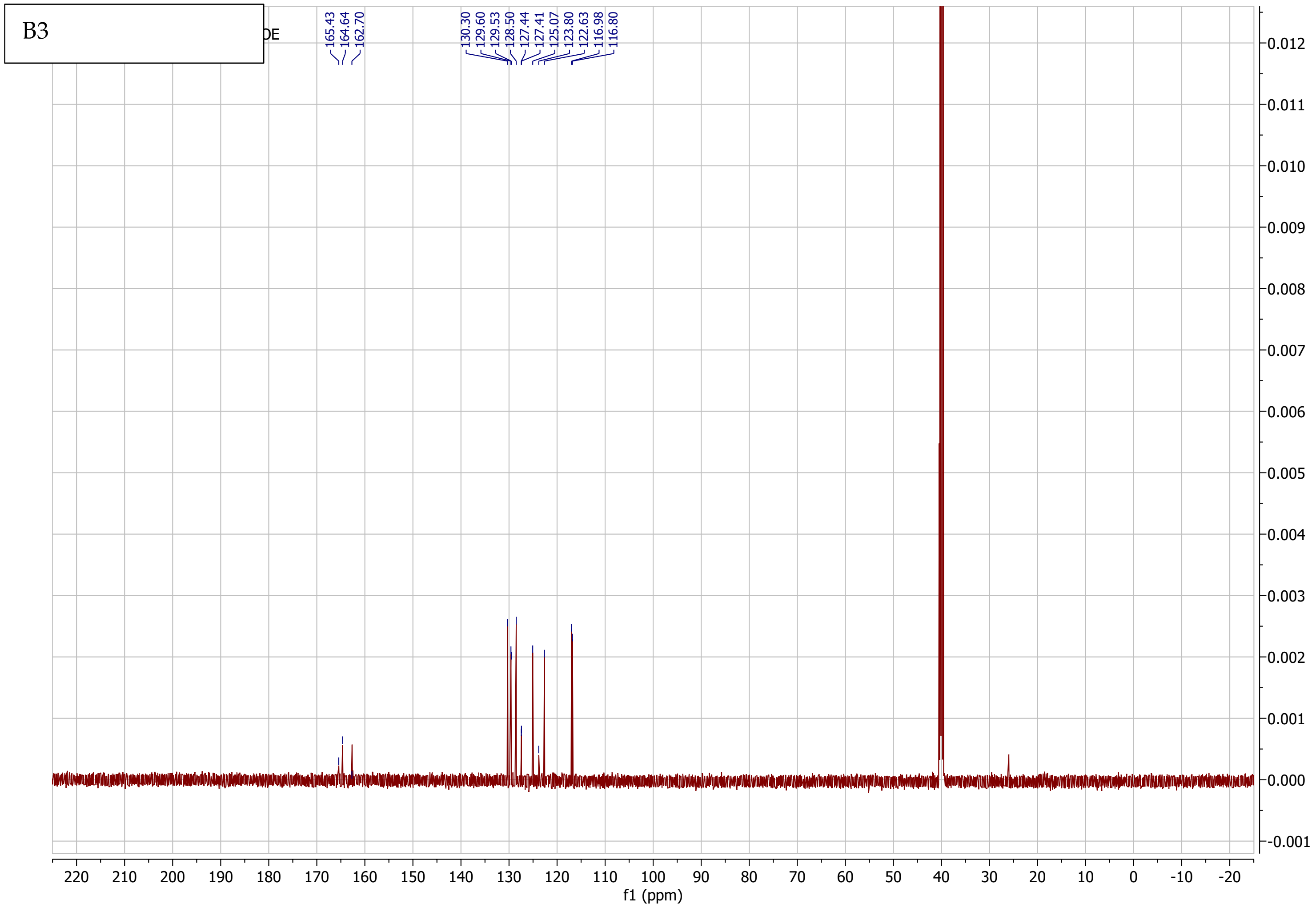

Figure S24. The ^{13}C NMR of compound B3.

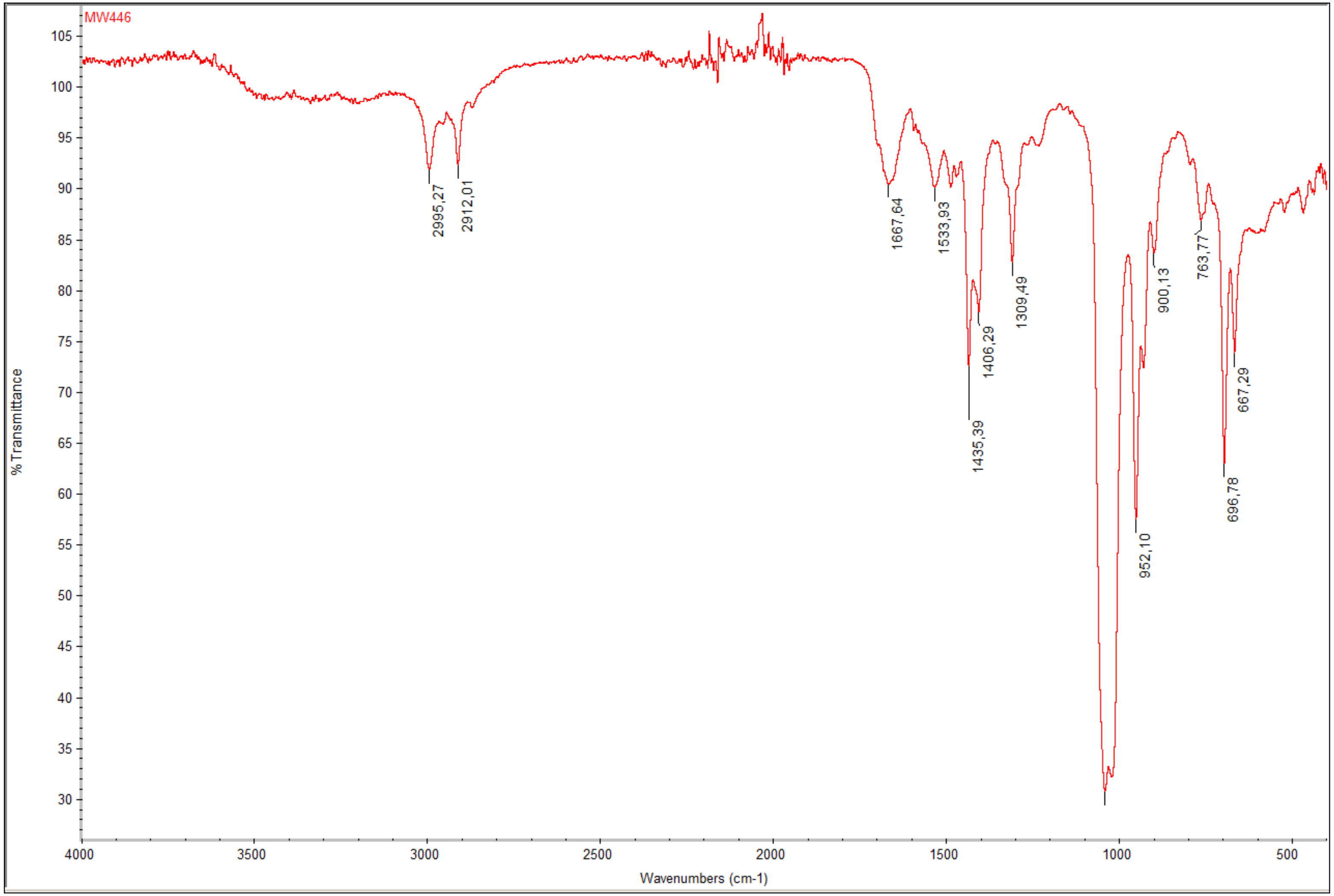

Figure S25. The IR of compound A1.

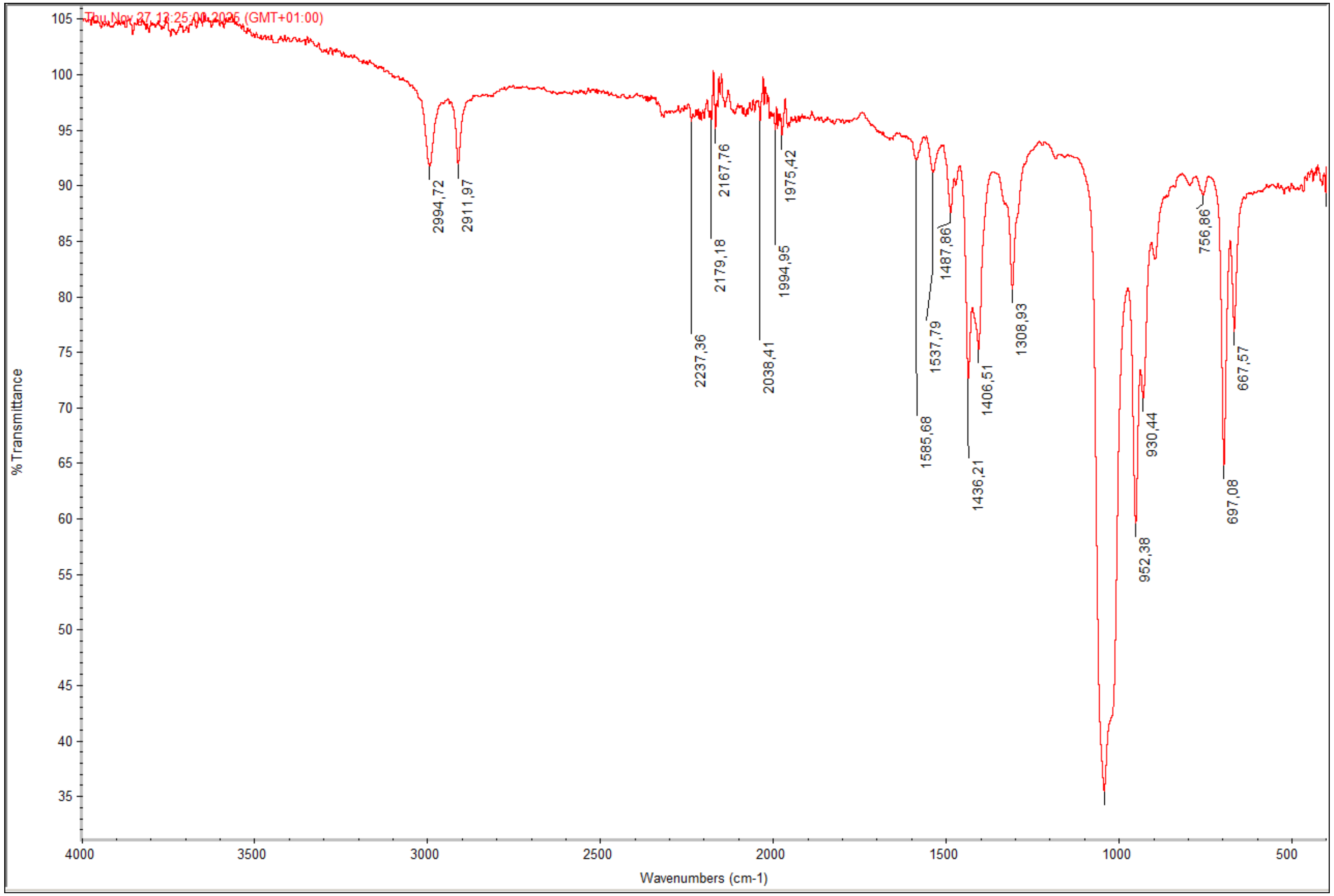

Figure S26. The IR of compound A2.

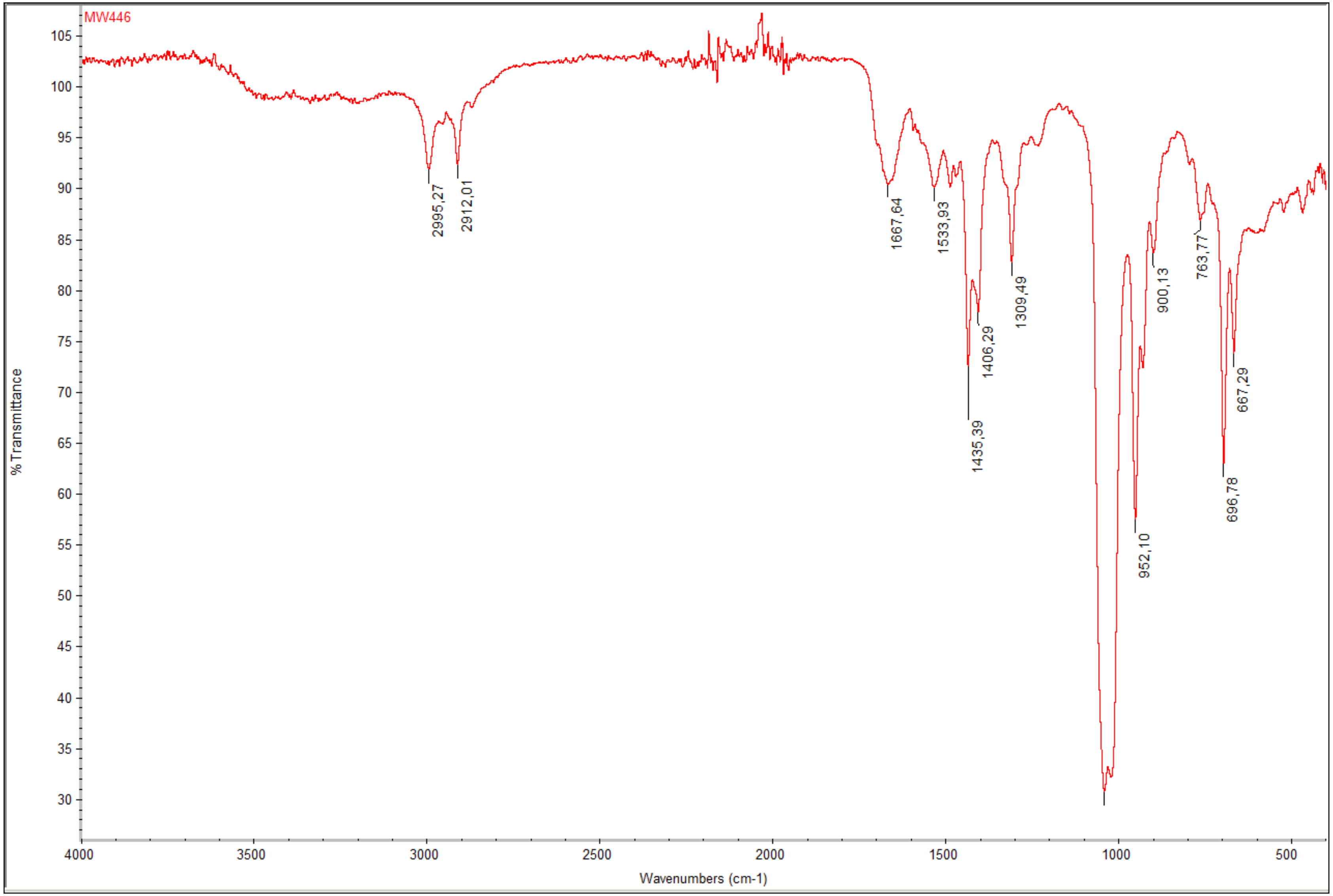

Figure S27. The IR of compound A3.

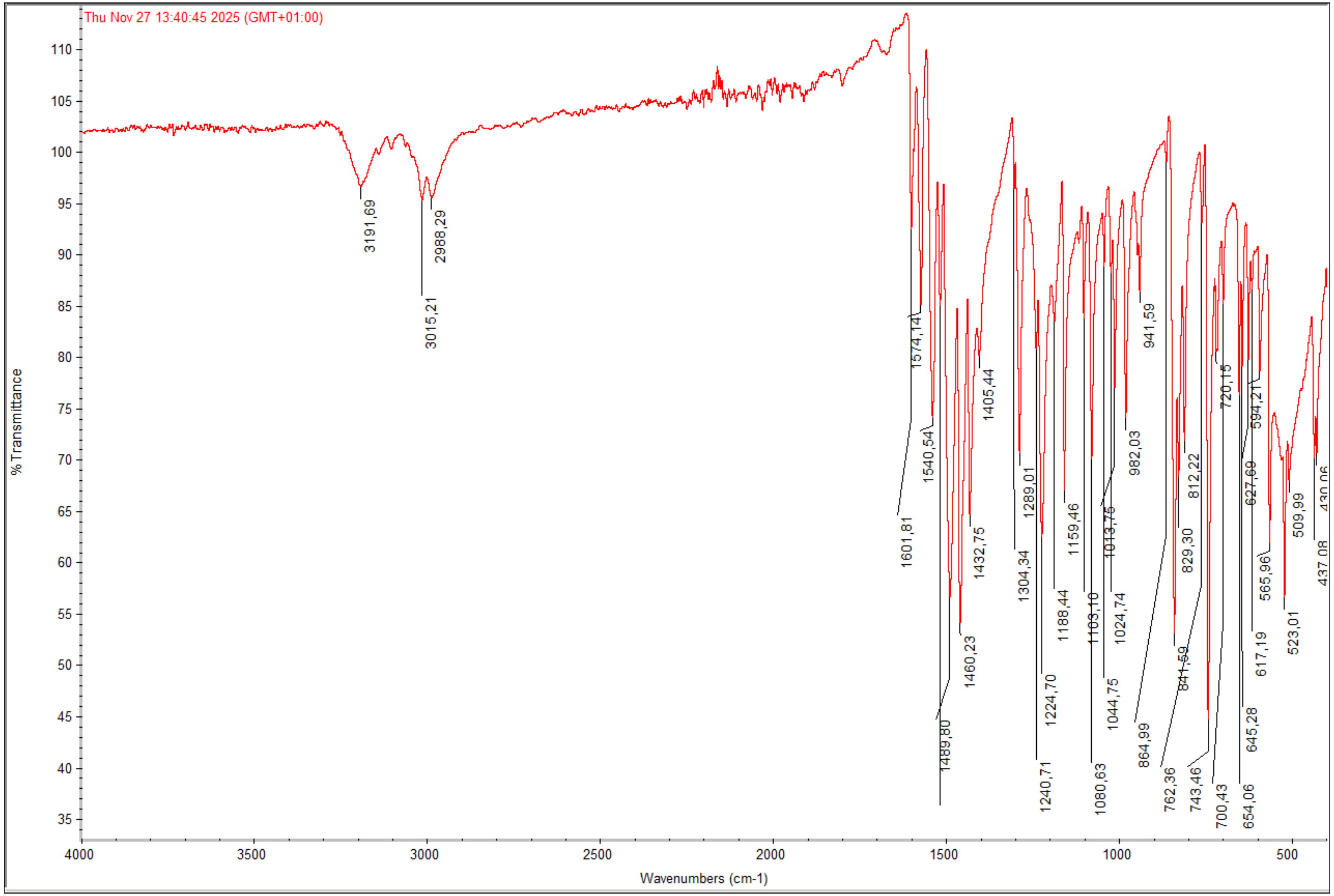

Figure S28. The IR of compound B1.

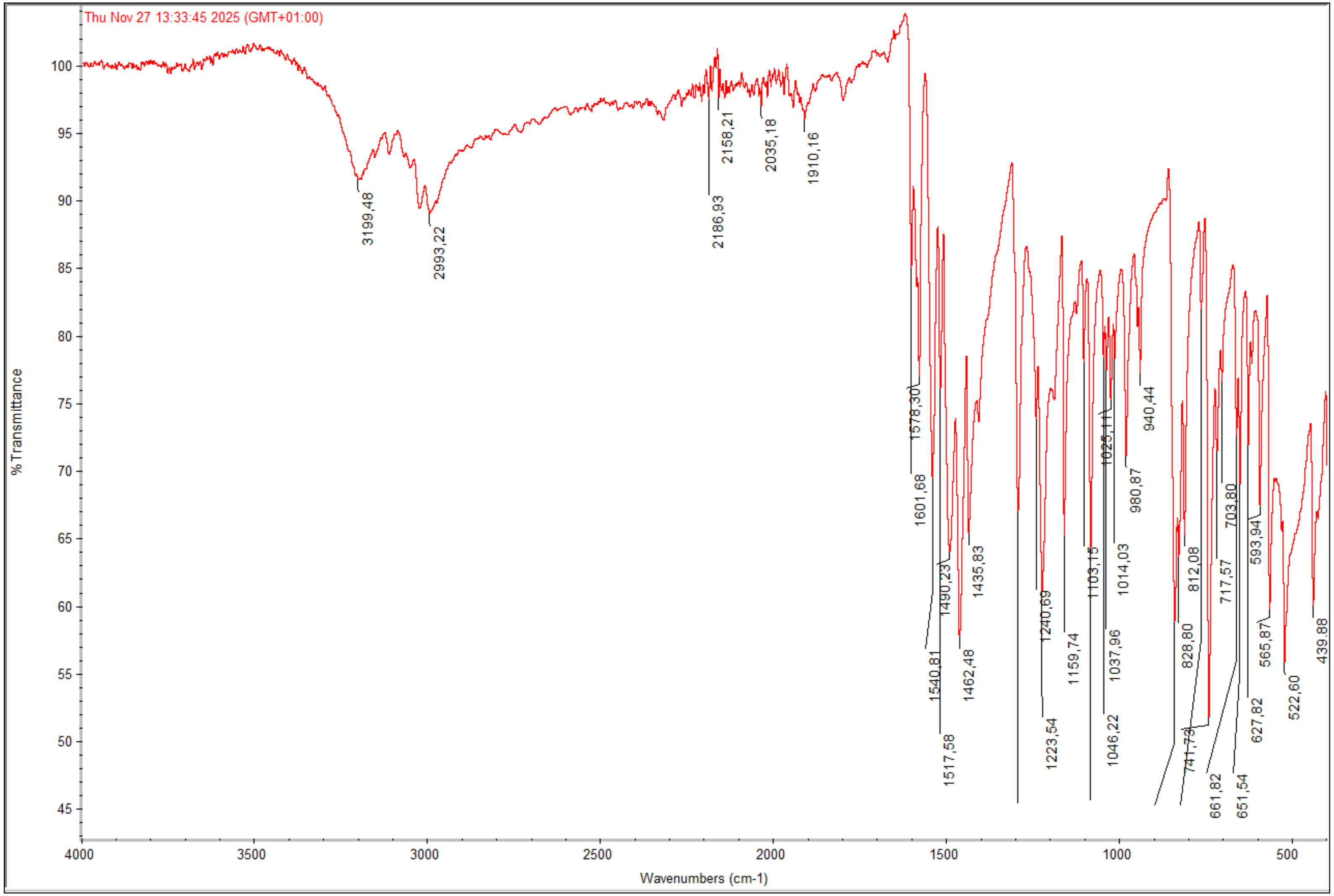

Figure S29. The IR of compound B2.

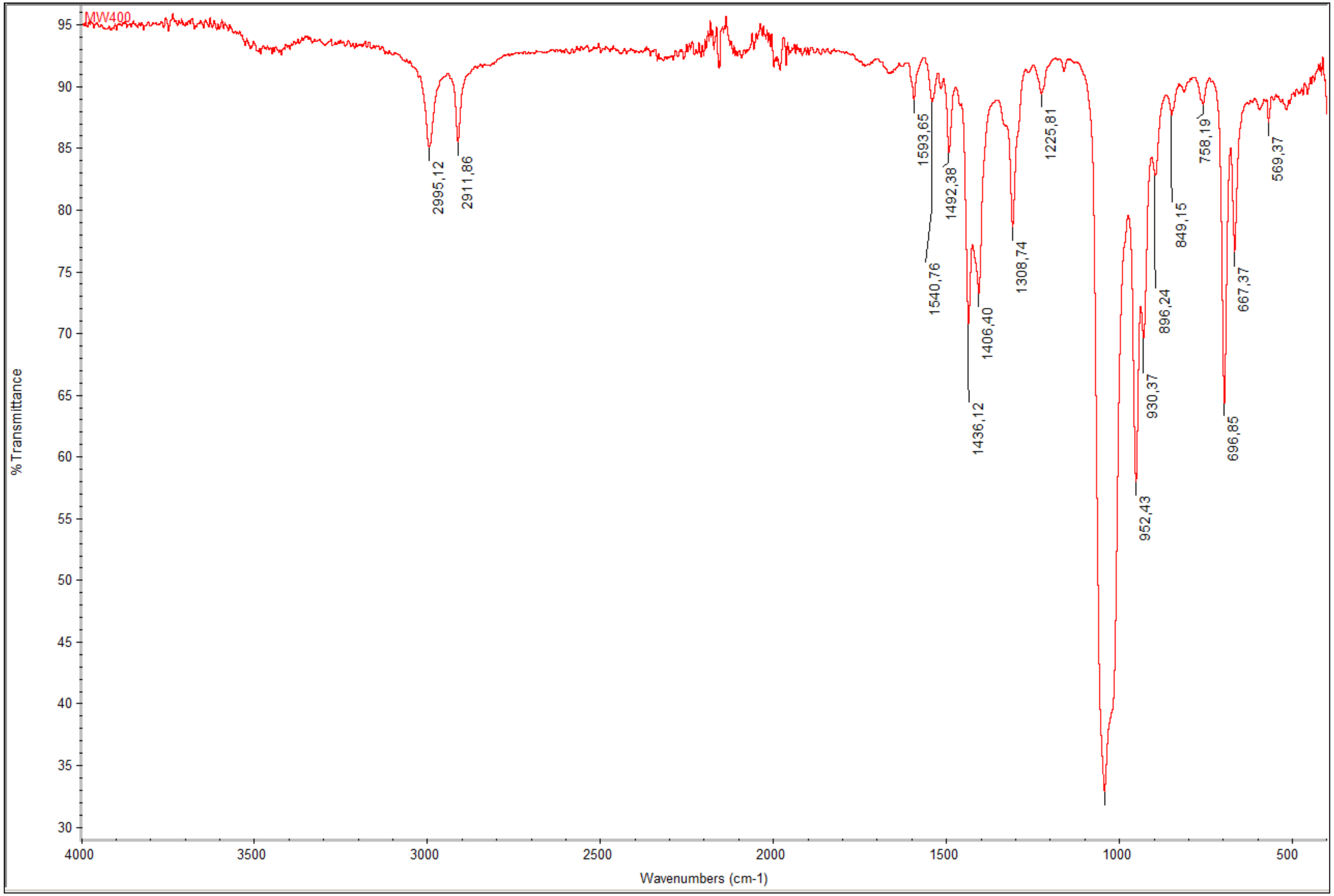

Figure S30. The IR of compound B3.

SHIMADZU LabSolutions

Analysis Report

Sample Name	: 1A
Sample ID	:
Data Filename	: SJ-446_MeOH_70-30m-03_(110-1500da)_1-12-2025_1.lcd
Method Filename	: MeOH_70-30m-03_(110-1500da).lcm
Batch Filename	: 1-12-2025.lcb
Vial #	: 3-38
Sample Type	: Unknown
Injection Volume	: 0.1 uL
Date Acquired	: 12/1/2025 4:58:34 PM
Acquired by	: System Administrator
Date Processed	: 12/1/2025 5:28:35 PM
Processed by	: System Administrator

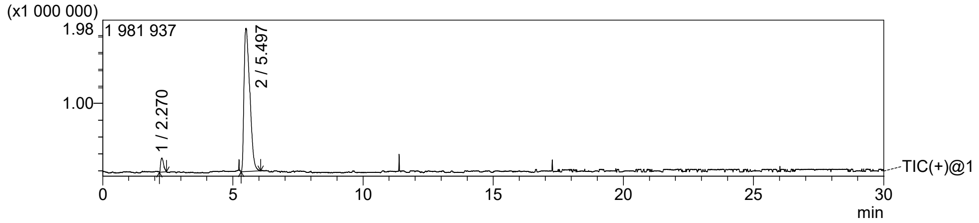

MASS Peak Table TIC

Peak#	Ret. Time	m/z	Area%
1	2.270	TIC	4.753
2	5.497	TIC	95.247
Total			100.000

MS Spectrum

Line#:1 R.Time:----(Scan#:----)
MassPeaks:163
Spectrum Mode:Averaged 2.265-2.275(454-456) Base Peak:157(56657)
BG Mode:Calc Segment 1 - Event 1

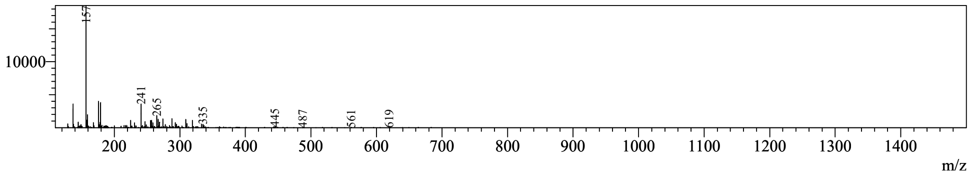

Line#:2 R.Time:----(Scan#:----)
MassPeaks:178
Spectrum Mode:Averaged 5.490-5.500(1099-1101) Base Peak:398(426922)
BG Mode:Calc Segment 1 - Event 1

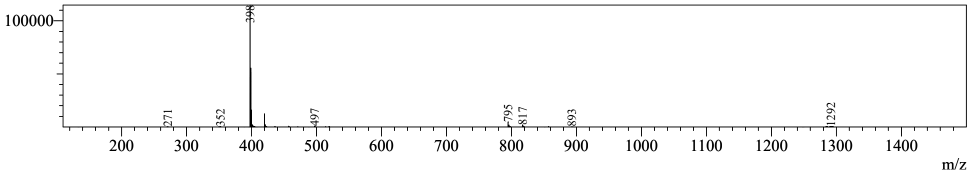

Figure S31. The MS of compound 1A.

Analysis Report

SHIMADZU LabSolutions

Sample Name	: [illegible]		
Sample ID	: 2A		
Data Filename	: SJ-435_MeOH_70-30m-03_(110-1500da)_1-12-2025_2.lcd		
Method Filename	: MeOH_70-30m-03_(110-1500da).lcm		
Batch Filename	: 1-12-2025.lcb		
Vial #	: 3-37	Sample Type	: Unknown
Injection Volume	: 0.1 uL		
Date Acquired	: 12/1/2025 5:29:01 PM	Acquired by	: System Administrator
Date Processed	: 12/1/2025 5:59:02 PM	Processed by	: System Administrator

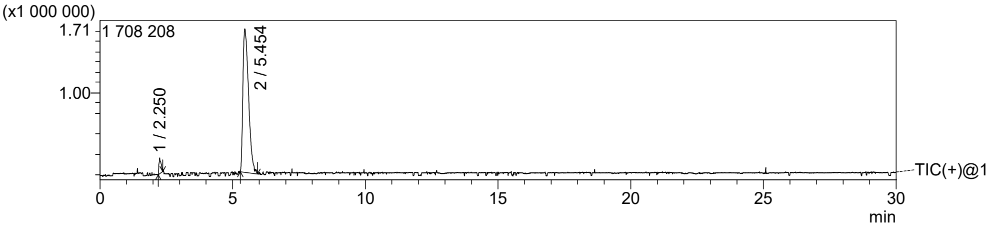

MASS Peak Table TIC

Peak#	Ret. Time	m/z	Area%
1	2.250	TIC	3.765
2	5.454	TIC	96.235
Total			100.000

MS Spectrum

Line#:1 R.Time:----(Scan#:----)
MassPeaks:177
Spectrum Mode:Averaged 2.245-2.255(450-452) Base Peak:157(46447)
BG Mode:Calc Segment 1 - Event 1

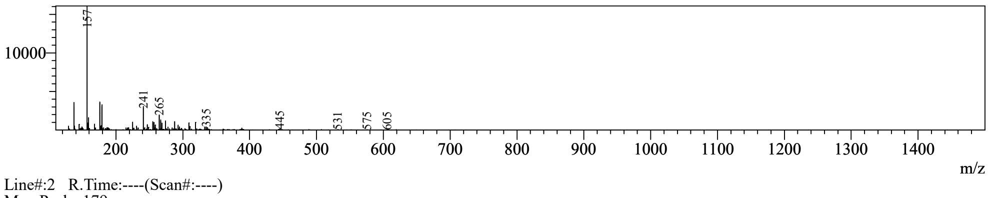

Line#:2 R.Time:----(Scan#:----)
MassPeaks:179
Spectrum Mode:Averaged 5.445-5.455(1090-1092) Base Peak:352(467442)
BG Mode:Calc Segment 1 - Event 1

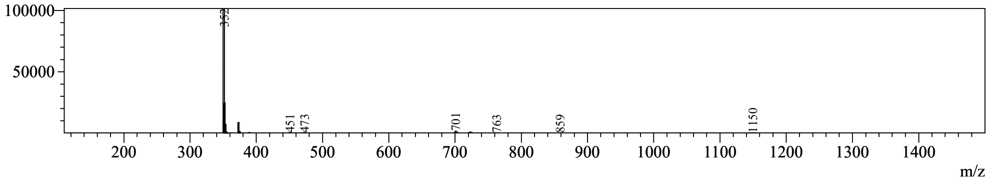

Figure S32. The MS of compound 2A.

Analysis Report

SHIMADZU LabSolutions

Sample Name	: 3A
Sample ID	:
Data Filename	: SJ-434_MeOH_70-30m-03_(110-1500da)_1-12-2025_2.lcd
Method Filename	: MeOH_70-30m-03_(110-1500da).lcm
Batch Filename	: 1-12-2025.lcb
Vial #	: 3-40
Injection Volume	: 0.1 uL
Date Acquired	: 12/1/2025 6:29:52 PM
Date Processed	: 12/2/2025 1:34:10 PM
Sample Type	: Unknown
Acquired by	: System Administrator
Processed by	: System Administrator

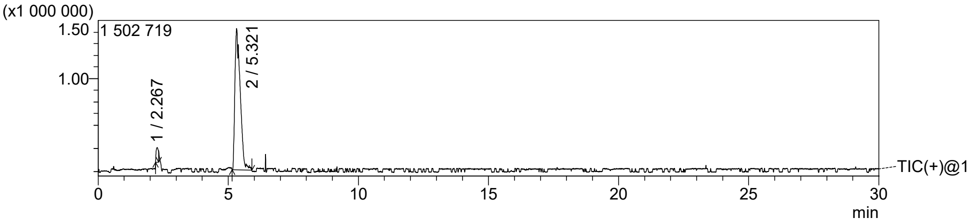

MASS Peak Table TIC

Peak#	Ret. Time	m/z	Area%
1	2.267	TIC	3.972
2	5.321	TIC	96.028
Total			100.000

MS Spectrum

Line#:1 R.Time:----(Scan#:----)
MassPeaks:213
Spectrum Mode:Averaged 5.315-5.325(1064-1066) Base Peak:306(121705)
BG Mode:Calc Segment 1 - Event 1

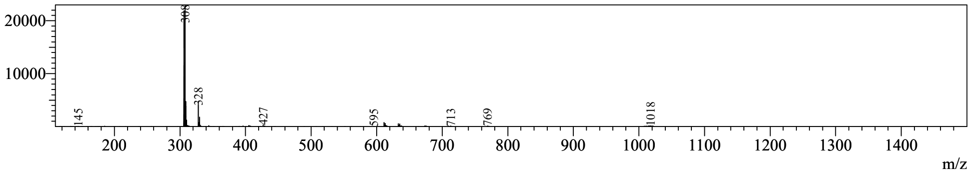

Line#:2 R.Time:----(Scan#:----)
MassPeaks:249
Spectrum Mode:Averaged 5.375-5.385(1076-1078) Base Peak:306(30878)
BG Mode:Calc Segment 1 - Event 1

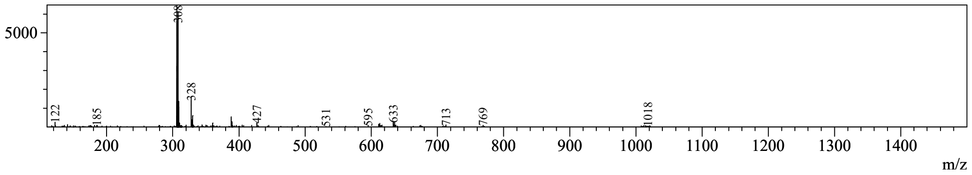

Figure S33. The MS of compound 3A.

SHIMADZU LabSolutions

Analysis Report

Sample Name : 1B
Sample ID :
Data Filename : SJ-4f2jt_MeOH_70-30m-03_(110-1500da)_2-12-2025_3.lcd
Method Filename : MeOH_70-30m-03_(110-1500da).lcm
Batch Filename : 2-12-2025.lcb
Vial # : 3-42
Sample Type : Unknown
Injection Volume : 0.2 uL
Date Acquired : 12/2/2025 10:57:48 AM
Acquired by : System Administrator
Date Processed : 12/2/2025 11:27:49 AM
Processed by : System Administrator

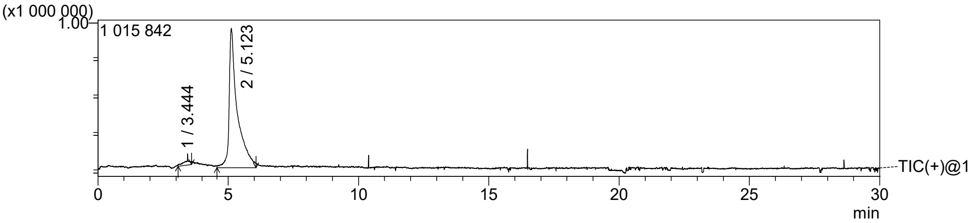

MASS Peak Table TIC

Peak#	Ret. Time	m/z	Area%
1	3.444	TIC	2.906
2	5.123	TIC	97.094
Total			100.000

MS Spectrum

Line#:1 R.Time:----(Scan#:----)
MassPeaks:220
Spectrum Mode:Averaged 3.435-3.445(688-690) Base Peak:297(16856)
BG Mode:Calc Segment 1 - Event 1

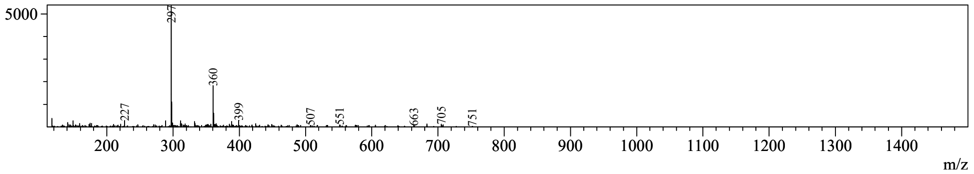

Line#:2 R.Time:----(Scan#:----)
MassPeaks:146
Spectrum Mode:Averaged 5.115-5.125(1024-1026) Base Peak:398(408005)
BG Mode:Calc Segment 1 - Event 1

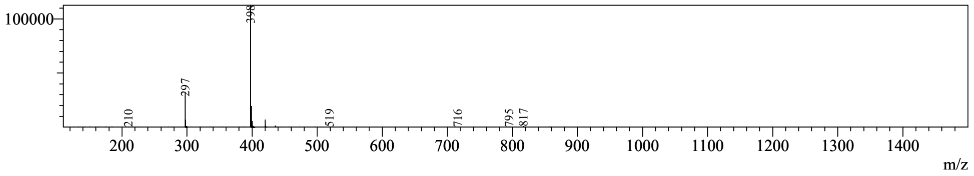

Figure S34. The MS of compound 1B.

SHIMADZU LabSolutions

Analysis Report

Sample Name	2B
Sample ID	:
Data Filename	: SJ-377_MeOH_70-30m-03_(110-1500da)_1-12-2025_4.lcd
Method Filename	: MeOH_70-30m-03_(110-1500da).lcm
Batch Filename	: 1-12-2025.lcb
Vial #	: 3-41
Injection Volume	: 0.1 uL
Date Acquired	: 12/1/2025 7:30:42 PM
Date Processed	: 12/1/2025 8:00:43 PM
Sample Type	: Unknown
Acquired by	: System Administrator
Processed by	: System Administrator

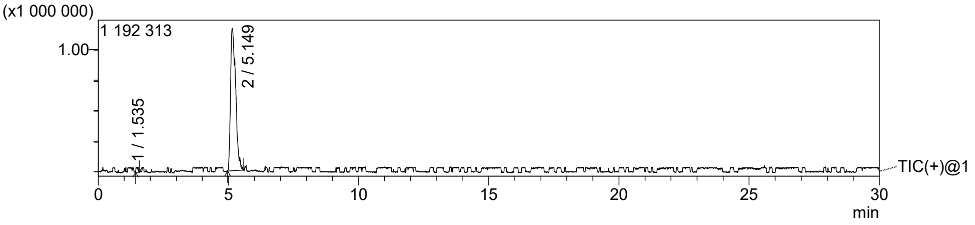

MASS Peak Table TIC

Peak#	Ret. Time	m/z	Area%
1	1.535	TIC	1.484
2	5.149	TIC	98.516
Total			100.000

MS Spectrum

Line#:1 R.Time:----(Scan#:----)
MassPeaks:163
Spectrum Mode:Averaged 1.530-1.540(307-309) Base Peak:388(4923)
BG Mode:Calc Segment 1 - Event 1

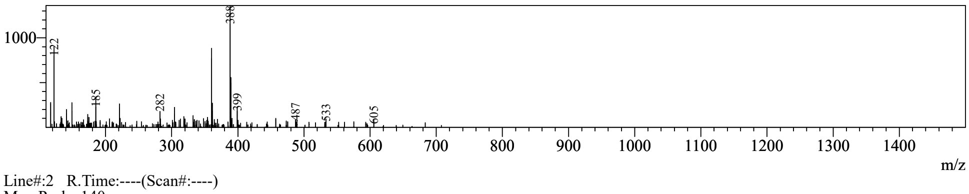

Line#:2 R.Time:----(Scan#:----)
MassPeaks:140
Spectrum Mode:Averaged 5.140-5.150(1029-1031) Base Peak:352(331372)
BG Mode:Calc Segment 1 - Event 1

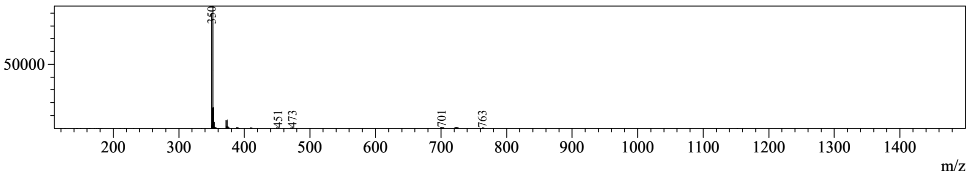

Figure S35. The MS of compound 2B.

Analysis Report

SHIMADZU LabSolutions

Sample Name : 3B
Sample ID :
Data Filename : SJ-400_MeOH_70-30m-03_(110-1500da)_1-12-2025_1.lcd
Method Filename : MeOH_70-30m-03_(110-1500da).lcm
Batch Filename : 1-12-2025.lcb
Vial # : 3-39 Sample Type : Unknown
Injection Volume : 0.1 uL
Date Acquired : 12/1/2025 5:59:26 PM Acquired by : System Administrator
Date Processed : 12/2/2025 1:36:18 PM Processed by : System Administrator

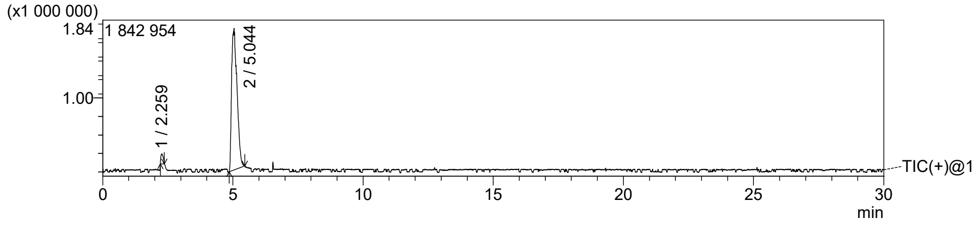

MASS Peak Table TIC

Peak#	Ret. Time	m/z	Area%
1	2.259	TIC	2.992
2	5.044	TIC	97.008
Total			100.000

MS Spectrum

Line#:1 R.Time:----(Scan#:----)
MassPeaks:191
Spectrum Mode:Averaged 2.250-2.260(451-453) Base Peak:157(58178)
BG Mode:Calc Segment 1 - Event 1

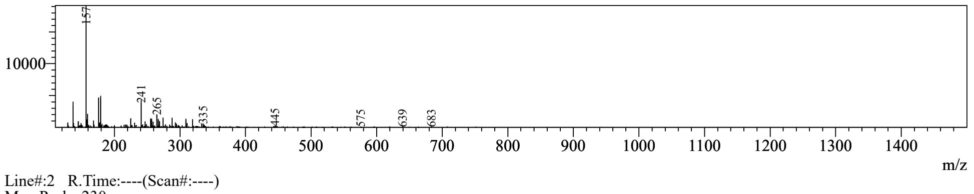

Line#:2 R.Time:----(Scan#:----)
MassPeaks:230
Spectrum Mode:Averaged 5.035-5.045(1008-1010) Base Peak:306(478893)
BG Mode:Calc Segment 1 - Event 1

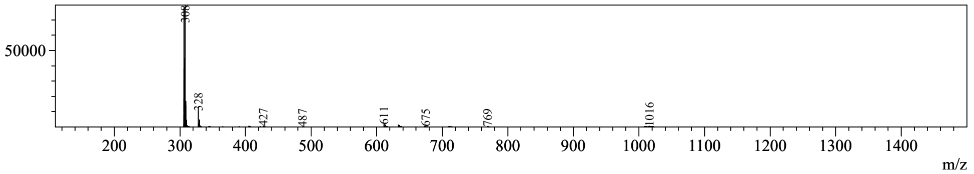

Figure S36. The MS of compound 3B

Molecular Dynamics Analysis of Binding Modes of Compound B3

Analysis of the MD trajectories provided an insight into the dynamic stability of the three binding modes of compound **B3**, selected based on the molecular docking. The time evolution of the trajectories revealed that the first binding mode (green, Figure S37) maintained its orientation throughout the entire simulation, exhibiting only minimal fluctuations. In contrast, the second binding mode (magenta) underwent a temporary reorientation of the 2-Cl–Ar fragment around 110 ns, but reverted to its original orientation after approximately 30 ns, suggesting partial flexibility within the binding pocket. The third binding mode, which exhibited the highest average RMSD, showed a distinct and irreversible flip of the 2-Cl–Ar group at around 100 ns, maintaining this altered orientation until the end of the simulation.

These findings confirm that only the first conformation of compound **B3** remains stably anchored within the active site of aromatase, consistent with the interactions identified in the docking analysis. The transient rearrangements observed for the other poses suggest that alternative binding orientations are energetically less favorable under physiological conditions.

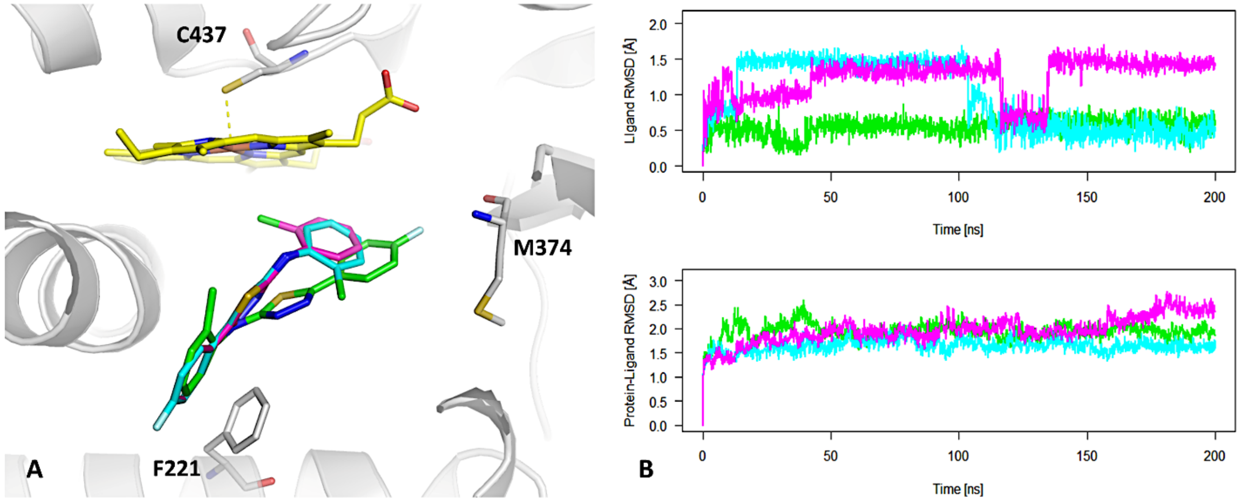

Figure S37. (A) Illustration of three competing binding modes of compound **B3** obtained from molecular docking analysis within the aromatase active site. (B) Results of 200 ns molecular dynamics (MD) simulations for the three selected binding modes of compound **B3**, showing RMSD plots for the ligand and the ligand–receptor complexes (line colors correspond to the ligand conformations depicted in panel A).
